# Spatial transcriptomics reveals the distinct organization of mouse prefrontal cortex and neuronal subtypes regulating chronic pain

**DOI:** 10.1038/s41593-023-01455-9

**Published:** 2023-10-16

**Authors:** Aritra Bhattacherjee, Chao Zhang, Brianna R. Watson, Mohamed Nadhir Djekidel, Jeffrey R. Moffitt, Yi Zhang

**Affiliations:** 1 Howard Hughes Medical Institute, Boston Children’s Hospital, Boston, MA USA; 2 Program in Cellular and Molecular Medicine, Boston Children’s Hospital, Boston, MA USA; 3 Division of Hematology/Oncology, Department of Pediatrics, Boston Children’s Hospital, Boston, MA USA; 4 Department of Microbiology, Harvard Medical School, Boston, MA USA; 5 Center for Applied Bioinformatics, St. Jude Children’s Research Hospital, Memphis, TN USA; 6 Department of Genetics, Harvard Medical School, Boston, MA USA; 7 Harvard Stem Cell Institute, Boston, MA USA

**Keywords:** Molecular neuroscience, Cellular neuroscience

## Abstract

The prefrontal cortex (PFC) is a complex brain region that regulates diverse functions ranging from cognition, emotion and executive action to even pain processing. To decode the cellular and circuit organization of such diverse functions, we employed spatially resolved single-cell transcriptome profiling of the adult mouse PFC. Results revealed that PFC has distinct cell-type composition and gene-expression patterns relative to neighboring cortical areas—with neuronal excitability-regulating genes differently expressed. These cellular and molecular features are further segregated within PFC subregions, alluding to the subregion-specificity of several PFC functions. PFC projects to major subcortical targets through combinations of neuronal subtypes, which emerge in a target-intrinsic fashion. Finally, based on these features, we identified distinct cell types and circuits in PFC underlying chronic pain, an escalating healthcare challenge with limited molecular understanding. Collectively, this comprehensive map will facilitate decoding of discrete molecular, cellular and circuit mechanisms underlying specific PFC functions in health and disease.

## Abstract (web summary)

Spatial transcriptomics reveals distinct composition and organization of cells and circuits in the mouse prefrontal cortex (PFC) relative to adjacent cortices, which concur with PFC’s diverse functions, and also help detect neurons involved in chronic pain.

## Main

The prefrontal cortex (PFC) is a major region of the mammalian brain that has evolved to perform highly complex behavioral functions. It plays important roles in cognition, emotion, reward and executive function. The PFC engages in complex executive tasks that dynamically coordinate cognition, attention, learning, memory, judgment, etc. to direct the action of an organism^1,2^. As such, dysfunctions of the PFC are associated with many cognitive and neuropsychiatric disorders^3,4^.

In addition to regulating intellectual and emotional behaviors, PFC is even involved in modulating pain processing as well as the negative effect of pain^5,6^. Increasing evidence indicates that disruption of this regulation is associated with the development of chronic pain, a rapidly increasing healthcare challenge that affects about 20% of the US population with economic burdens exceeding that of diabetes or heart disease^7,8^. Chronic pain has been associated with PFC hypoactivity, and transcranial stimulation of the PFC can induce pain relief^9–13^. Although projections from PFC to brainstem have been historically described in descending inhibition of pain^5,14,15^, the underlying molecular mechanism is poorly understood. Besides, PFC interacts with many downstream targets including the amygdala, nucleus accumbens (NAc) and thalamus—the major components of the central pain matrix, critical for the sensory or affective symptoms of chronic pain^6,16^. As such, PFC has an important role in pain ‘chronification’^5,15^.

Thus, a central question is how does PFC organize and manage such diverse functions—from cognitive processes to autonomic pain modulation? To address this question, we and others have previously performed single-cell RNA-seq (scRNA-seq) to decode the cellular heterogeneity of the PFC^2,17^, which revealed a myriad of cell types. However, those studies lacked information about the spatial organization and interaction of the diverse cell types, which are major determinants of the functional diversity of the PFC. A relatively homogeneous histology, with a laminar organization, is the most striking feature of the mammalian cerebral cortex^18–20^. Yet, distinct regions of cortex perform highly specialized functions, including vision, locomotion and somatosensation. This regional specialization of functions, despite apparent homogeneity, is likely due to the distinct features at multiple levels, including molecular composition (transcriptome), circuit organization (connectome) and anatomical (spatial) organization of cell subtypes within each cortical area. Decoding such organizational logic is critical not only for mechanistic understanding of cortical function but also for developing drugs to selectively target neurological disorders of cortical origin, such that drugs directed to either cognitive (frontal cortex) or hearing (auditory cortex) defect do not disrupt visual or motor function.

Approaching such questions has been historically limited by technological barriers, despite extensive scRNA-seq profiling across the brain, including cortex^21–23^. With recent advances in spatial transcriptomics techniques, such questions can now be addressed. Using multiplexed error-robust fluorescence in situ hybridization (MERFISH), an image-based method for spatially resolved single-cell transcriptomics^24^, here we decode the spatial organization of the PFC and its subregions. Our results demonstrate distinct cellular composition of the PFC relative to its adjoining cortical areas. PFC adopts distinct molecular features to suit its specific electrophysiological properties, different from its adjacent cortices. We map molecular identities (and layer localization) of PFC’s projection neurons to major subcortical targets. Finally, based on projection, transcription and activity markers, we reveal the molecular identity of PFC neuronal clusters most substantially affected in chronic pain.

## Results

### Diversity and organization of cell types in mouse frontal cortex

**Extended Data Fig. 1:**
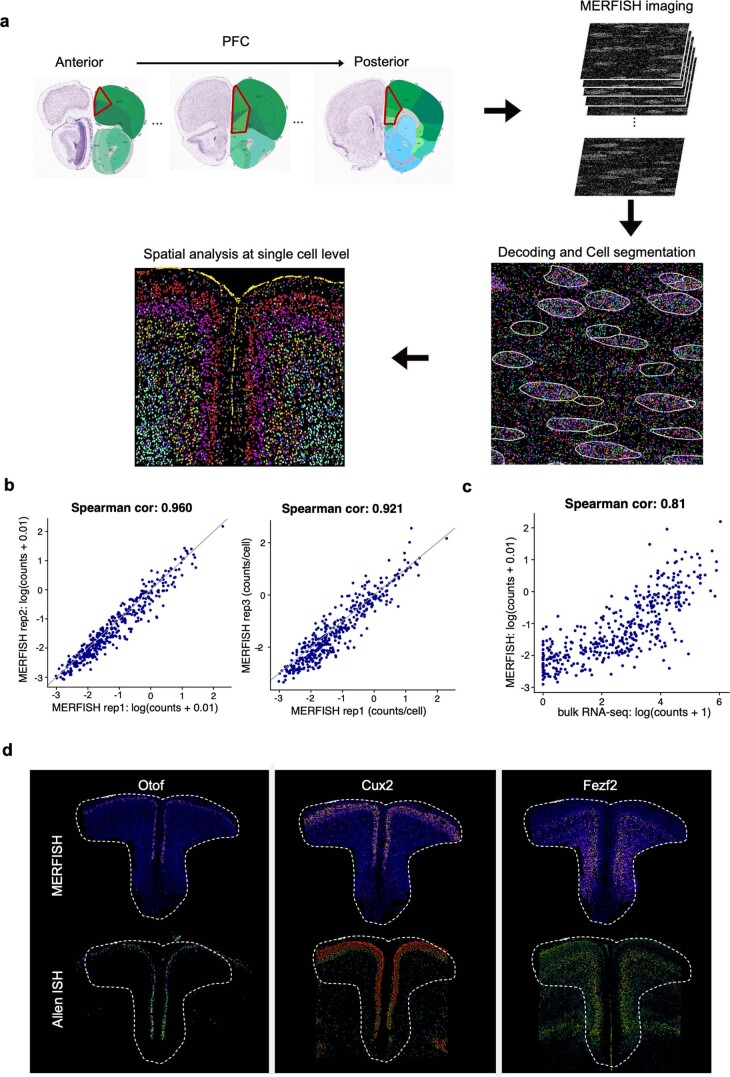
The workflow and quality control for MERFISH profiling. **a**, The workflow of MERFISH profiling of mouse PFC, including MERFISH imaging, decoding, segmentation and data analysis. **b**, Scatterplot showing the spearman correlation of the RNA counts per cell of individual genes measured by MERFISH in two independent experiments. **c**, Scatterplot of the RNA counts per cell of individual genes measured by MERFISH versus bulk RNA-seq data. The counts are natural logarithms. **d**, Spatial gene expression of three representative genes detected by MERFISH. In situ hybridization (ISH) data from Allen Brain Atlas are shown at the bottom. Source data

All experiments reported in this entire study were conducted in adult male mice. To understand the diversity of cell types and determine their spatial organization within the PFC, we performed MERFISH^24,25^, an imaging-based method for single-cell transcriptomics with error-robust barcoding read through iterative rounds of single-molecule FISH. MERFISH detects the precise location of each RNA molecule to ultimately reveal the spatial organization of diverse cell types within anatomically defined tissue regions (Extended Data Fig. 1a)^25,26^. The MERFISH library comprised 416 genes including cell-type markers and functionally important genes like ion channels, neuropeptides, G-protein coupled receptors and a panel of neuronal activity-regulated genes (ARG; Supplementary Table 1; see details in Methods). We collected brain samples from three different adult male mice and prepared rostral to caudal coronal slices covering +2.5 to +1.3 from Bregma to broadly image the frontal cortex. The imaged RNA species were detected, decoded and assigned to individual cells using established analysis pipelines^25^ (Extended Data Fig. 1a). Overall, we obtained 487,224 high-quality cells in the frontal cortical region from three independent biological replicates with high consistency (Extended Data Fig. 1b). Expression of individual genes showed good correlation with that of the bulk RNA-seq of the PFC (Extended Data Fig. 1c).

**Fig. 1:**
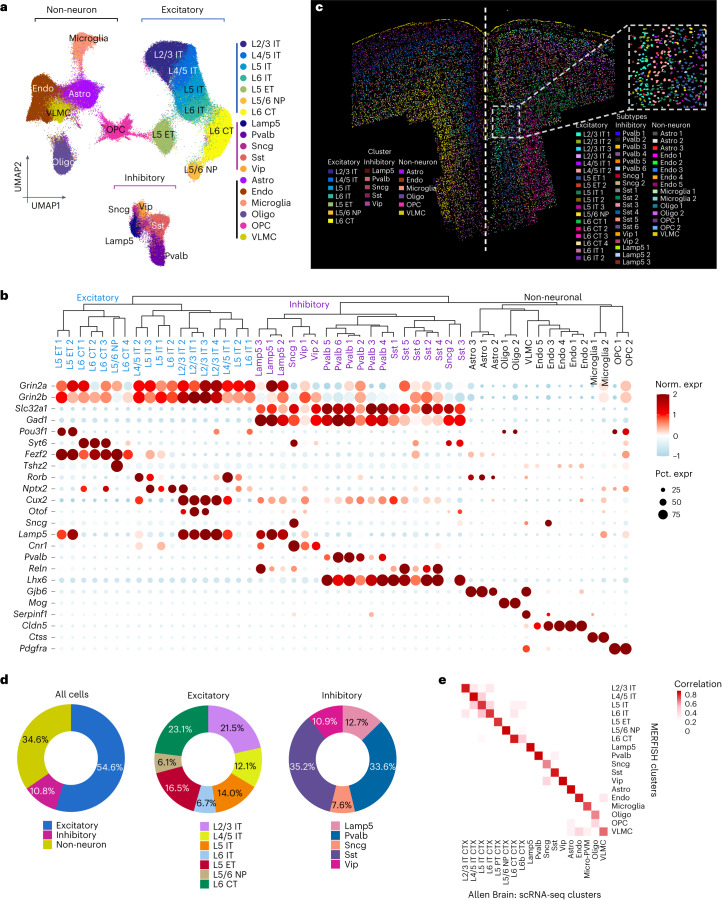
MERFISH reveals the molecularly diverse cell types and subtypes comprising the PFC and adjoining cortices. **a**, UMAP visualization of all cells identified by MERFISH. Cells are color-coded by their identities (number of cells = 487,224). **b**, Dendrogram showing the hierarchical relationship among all molecularly defined cell subtypes (number of cells = 487,224). The expression of marker genes is shown below. The color represents the normalized expression, and the dot size indicates the percentage of cells expressing each gene. **c**, Spatial map of all cell subtypes in a represented coronal slice. An enlarged view of a zoom-in region is shown in the top-right inset. **d**, Pie charts showing the cell proportions of the major cell types (left), excitatory neurons (middle) and inhibitory neurons (right) in PFC. **e**, Heatmap showing the gene-expression correlation between cell types and subtypes defined by MERFISH and scRNA-seq. scRNA-seq data were downloaded from Allen Brain Atlas, and only cells from PFC were used. Source data

Unsupervised clustering revealed the major cell types—excitatory neurons, inhibitory neurons and non-neuronal cells that include oligodendrocytes, oligodendrocyte precursors (OPC), microglia, endothelial cells, astrocytes and vascular leptomeningeal cells (VLMC) (Fig. 1a). Within the excitatory neurons, the major subgroups are clustered together, as described by the commonly used nomenclature^21^—the intratelencephalic (IT) populations of different layers, the extra-telencephalic (ET) neurons, the near projecting (NP) and the cortico-thalamic (CT) populations (Fig. 1a). Within the inhibitory neurons, populations from the medial ganglionic eminence (Pvalb and Sst) and the caudal ganglionic eminence (Vip, Sncg and Lamp5) clustered distinctively (Fig. 1a).

The major cell types were further clustered into the following 52 hierarchically organized cell subtypes: 18 excitatory, 19 inhibitory and 15 non-neuron cell subtypes (Fig. 1b). Four subtypes were detected in L2/3 IT (L2/3 IT 1 to L2/3 IT 4), two subtypes in L4/5, three subtypes in L5 and two subtypes in L6. Additionally, the L5 ET split into two subtypes, the L6 CT into four subtypes and L5/6 NP formed a single cluster. Among the inhibitory neurons, the Pvalb and Sst each split into six subtypes, the Lamp5 into three subtypes and the Vip and Sncg into two subtypes each (Fig. 1b). Among the non-neurons, the endothelial cells formed five subtypes, Endo1-5, while astrocytes formed three subtypes, and oligodendrocytes and OPCs each formed two subtypes.

Projecting these clusters in space (based on MERFISH coordinates) revealed the anatomical layout of the coronal section and precise localization of every single cell (Fig. 1c, inset—magnified view showing individual cells). Molecularly similar excitatory neurons localized together to form distinct layers, from which a laminar histology, characteristic of the cerebral cortex, emerged (L2/3 IT to L6 CT: outside inwards; Fig. 1c, left half). Within each layer, the subtypes are further organized in strata (for example, L2/3 IT 1 to L2/3 IT 4; Fig. 1c, right half—distribution of subtypes). Inhibitory neurons are broadly distributed and do not form specific layers, although some subtypes appear to be enriched within certain layers or subregions (Fig. 1c). Non-neuronal cells are also broadly distributed, except for enrichment of oligodendrocytes near the fiber tracts (for example, corpus callosum) and the VLMC in the outermost layer of the brain (Fig. 1c, yellow). Established markers like *Otof*, *Cux2* and *Fezf2*, respectively, localized to L2, L2/3 and L5 on the MERFISH slice, consistent with the in situ hybridization (ISH) images from Allen Brain Institute, further validated our analysis (Extended Data Fig. 1d).

Together, excitatory neurons comprise the largest population in PFC, followed by all non-neuronal cells combined and then the inhibitory neurons (Fig. 1d, left). Within excitatory neurons, the IT neurons are the largest subgroup, followed by the ET, NP and CT of deeper layers, respectively (Fig. 1d, middle). Within the inhibitory, Sst and Pvalb neurons are most abundant followed by the Lamp5, Scng and Vip (Fig. 1d, right).

**Extended Data Fig. 2:**
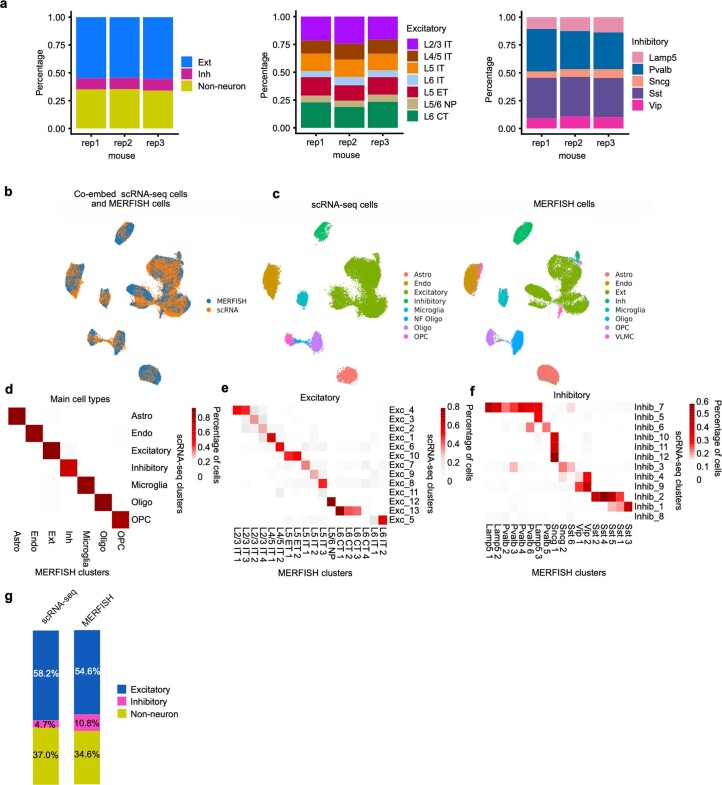
MERFISH and scRNA-seq based clusters are consistent. **a**, UMAP showing integration of cells from MERFISH or scRNA-seq data (GSE124952). **b**,**c**, UMAP showing the cell clusters defined by scRNA-seq (left) or MERFISH (right). **d**–**f**, Heatmap showing the correspondence between main cell types (d), excitatory (e) and inhibitory (f) subtypes defined by MERFISH and scRNA-seq. **g**, The cell proportions of the excitatory, inhibitory and non-neuronal cells from scRNA-seq or MERFISH. (Cell numbers for analyses in this figure, MERFISH = 121617, scRNAseq = 24822). Source data

There was no skewing between samples and similar percentages of cell types and subtypes were detected in the three biological replicates (Extended Data Fig. 2a). To further evaluate our detection accuracy, we first performed an integrated analysis of the MERFISH data with scRNA-seq data of the PFC from the Allen Institute^21^. All the major subtypes showed a strong correlation between the two datasets (Fig. 1e). Similar integrated analysis comparing the MERFISH data with our own scRNA-seq of PFC^17^ revealed strong correspondence even at the subtype levels (Extended Data Fig. 2b–f; Methods). In fact, MERFISH could classify some of the scRNA-seq clusters at a finer resolution to reveal distinct subclusters (Extended Data Fig. 2e,f). This point is particularly true for the inhibitory neurons (for example, Inh 1, 2 and 7 of scRNA-seq), possibly due to their higher rate of detection in MERFISH (Extended Data Fig. 2g).

**Extended Data Fig. 3:**
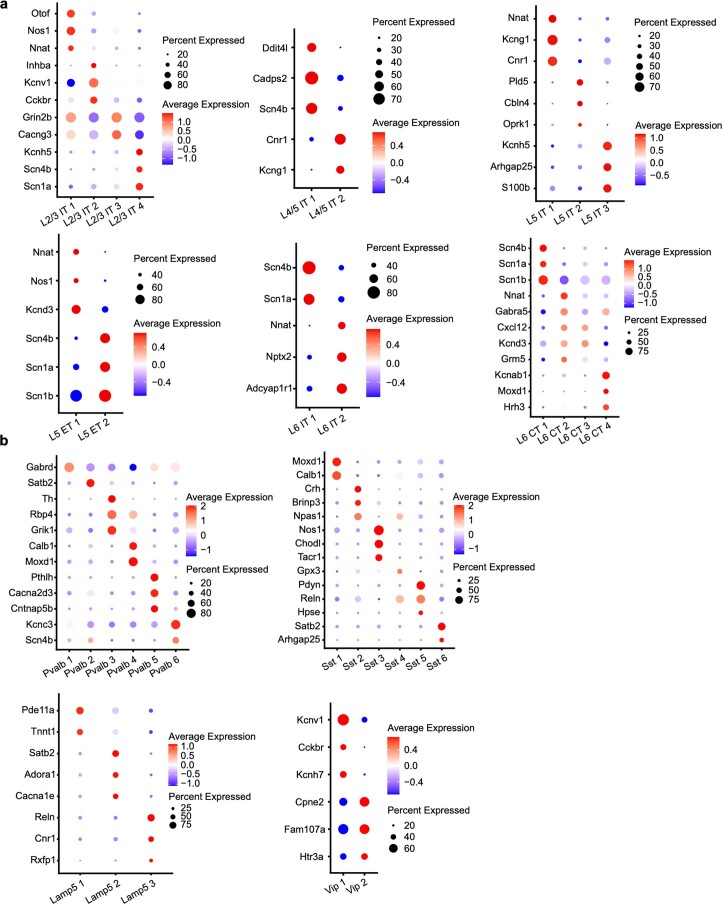
The marker gene expression in the different neuronal subtypes. **a**,**b**, Dot plot presentation for the top three marker genes for each of the excitatory (**a**) (Cell number = 247098) and inhibitory (**b**) (Cell number = 51794) neuron subtypes. Source data

**Extended Data Fig. 4:**
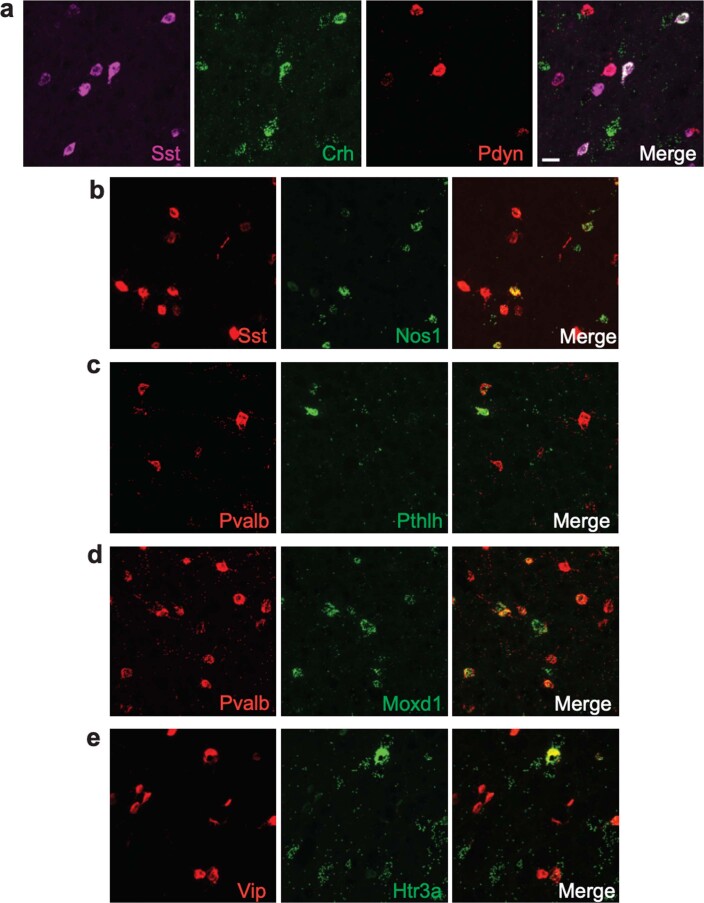
Single molecule FISH validates expression of inhibitory neuron markers and their overlap with several subtype-specific markers in PFC. **a**,**b**, smFISH for subtype-specific markers of Sst neurons. **c,d**, smFISH for subtype specific markers of Pvalb. **e**, Subtype specific marker that distinguishes the two identified Vip clusters. (Stains independently repeated 2 to 3 times). Scale bar for the figure in a = 20µm.

Further analysis revealed markers within each major group that can distinguish subtypes from each other in both excitatory (Extended Data Fig. 3a) and inhibitory (Extended Data Fig. 3b) neurons. Because inhibitory neurons are sparse and hence poorly understood, we validated some of their subtype markers using single-molecule FISH (RNAScope). Subclusters of each major cell type showed distinct marker expression (Extended Data Fig. 4). For example, *Crh* and *Pdyn*, selectively labeled Sst 2 and Sst 5 subclusters (Extended Data Fig. 4a), *Nos1* (Sst3 cluster marker) labeled only a subset of Sst neurons (Extended Data Fig. 4b), *Pthlh* and *Moxd1* (respective markers for Pvalb 5 and Pvalb 4) labeled respective subsets within Pvalb population (Extended Data Fig. 4c,d) and finally, *Htr3a* staining in Vip neurons revealed the classic *Htr3a*^*+*^ and *Htr3a*^*−*^ populations.

**Extended Data Fig. 5:**
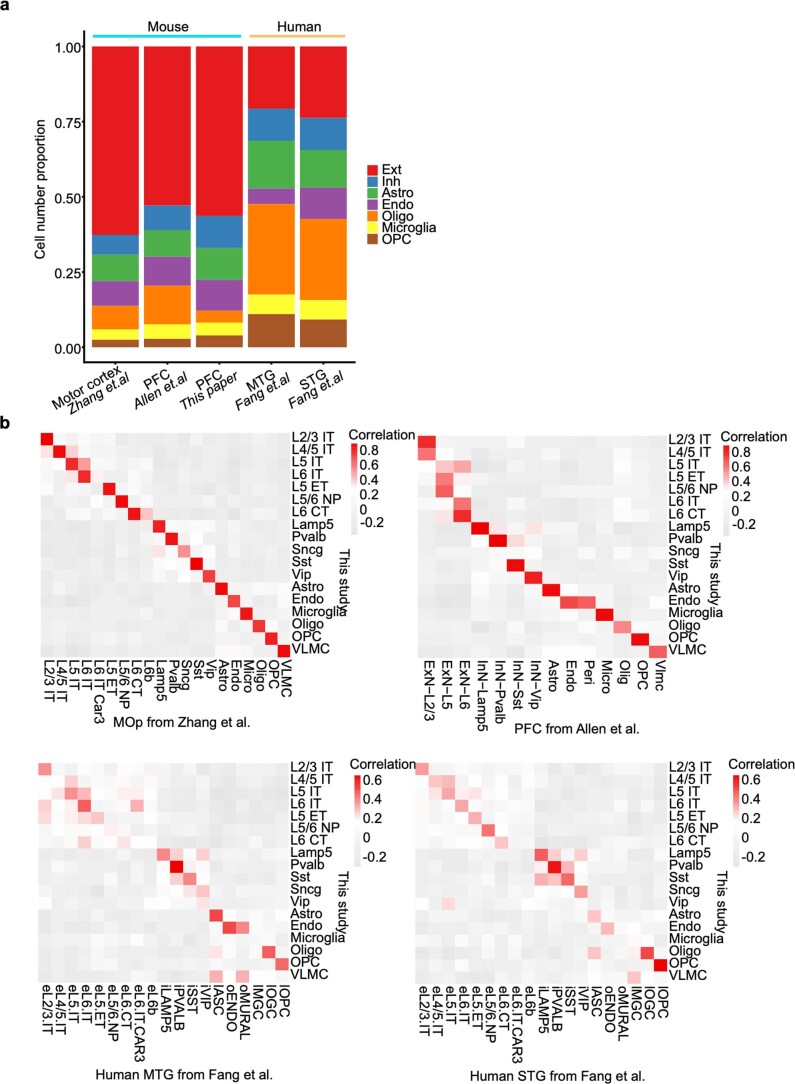
Comparative analysis between our dataset and those of published MERFISH dataset. **a**, Proportions of major cells types in ours compared with those in published papers. **b**, Heatmap showing the gene-expression correlation between cell types and subtypes defined in our study and those in published studies. (Cell numbers = motor cortex 23868; PFC_aging 185216; human_MTG 4321; human_STG 4871). Source data

To gain insights into the broader implications of our PFC profiling, we compared our PFC data with the following three recent MERFISH studies in mouse and human that covered parts of cortex: (1) mouse motor cortex (MOp) in ref. ^27^, (2) a snapshot area of mouse frontal brain (PFC/striatum/corpus callosum) in ref. ^28^ and (3) the superior and medial temporal gyri of human cortex in ref. ^29^. Cell composition appeared to be more closely related between the mouse studies than with that of human, with a higher proportion of non-neuronal cells in human (Extended Data Fig. 5a). Transcriptomic comparison also revealed strong correlation of subtypes with the mouse datasets, especially with MOp (Extended Data Fig. 5b). However, despite some conserved molecular signatures, human cortical neurons had a weaker correlation with the transcriptomic constitution of the mouse cells, particularly with MTG (Extended Data Fig. 5b). Thus, while the strong correlation across mouse datasets reaffirms our data quality and the power of MERFISH, a deeper profiling study is necessary to determine the precise correspondence of mouse and human cells, and how they diversified during evolution.

### Heterogeneous distribution of subtypes along A–P and D–V axes of mouse PFC

**Extended Data Fig. 6:**
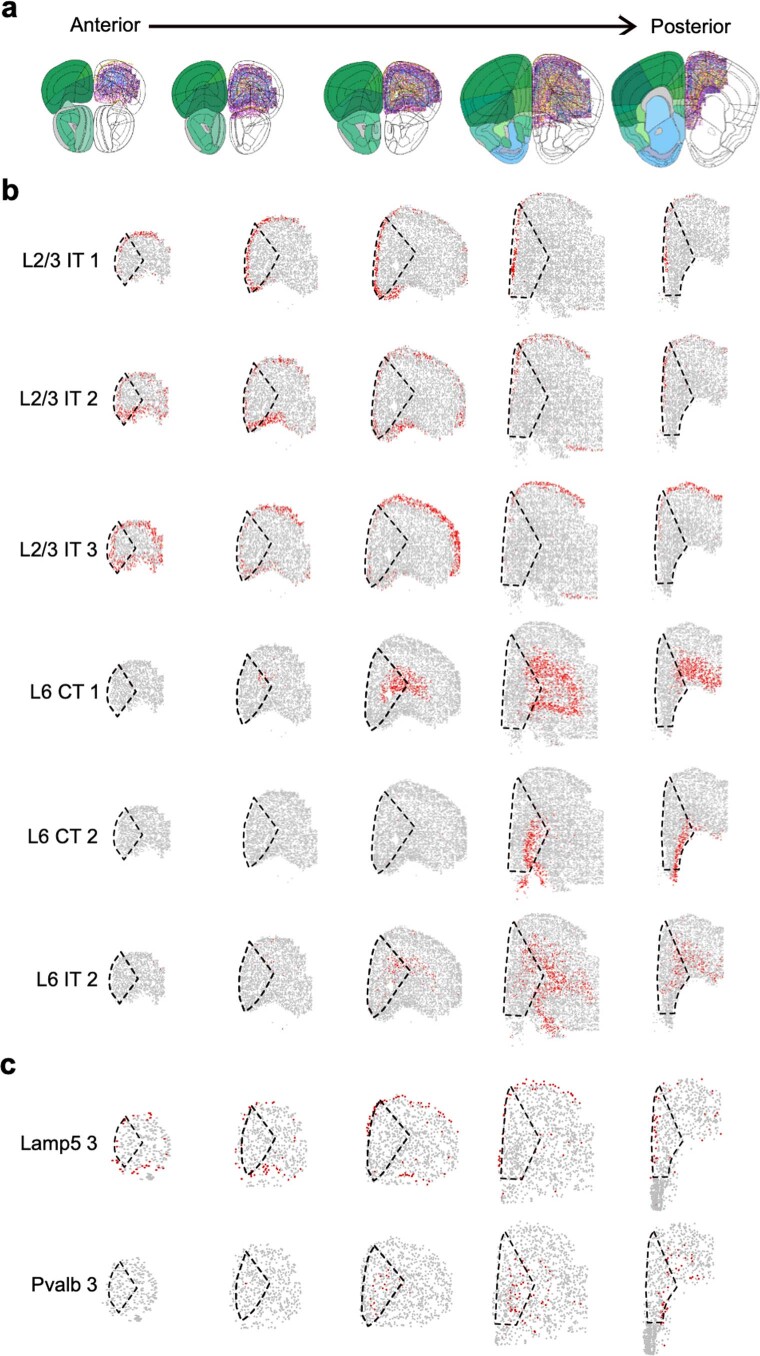
Spatial distribution of molecularly defined excitatory neuron subtypes along the anterior to posterior axis. **a**, Schematics of coronal brain slices aligned to Allen Brain Atlas CCF-v3 from anterior to posterior sections. **b**, Spatial organization of the indicated representative excitatory neuron subtypes across anterior to posterior sections. **c**, Spatial organization of the indicated representative inhibitory neuron subtypes across anterior to posterior sections.

To understand the spatial organization of the different neuron subtypes within the anatomically defined PFC region, we aligned our profiled serial sections with the Allen Mouse Brain Common Coordinate Framework (CCF) v3 (ref. ^30^), a reference created for the mouse brain based on serial two-photon tomography images of the 1675 C57Bl6/J mice (Extended Data Fig. 6a), which outlines the PFC boundaries within each section.

**Fig. 2:**
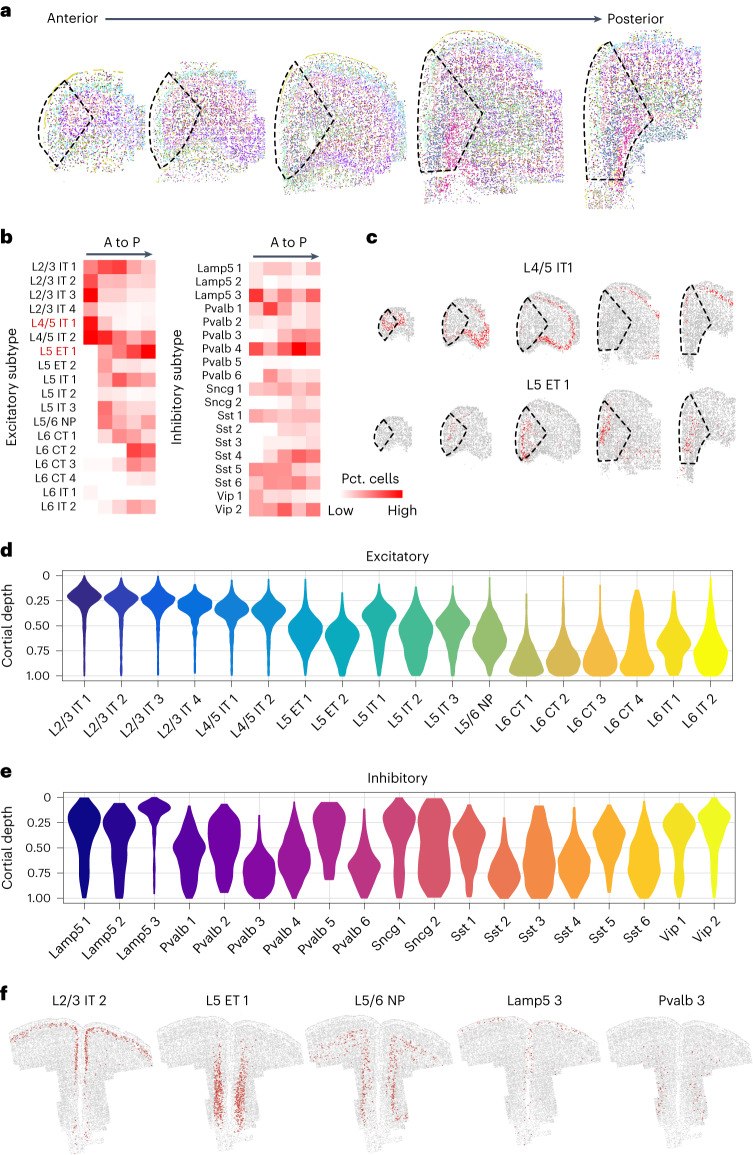
Spatial organization of different neuron subtypes in PFC. **a**, Coronal MERFISH slices showing the spatial organization of neuron subtypes from anterior to posterior end in PFC and adjacent regions. The dotted lines indicate the PFC region. The color scheme is the same as in Fig. 1c. **b**, Heatmap showing the proportions of neuron subtypes within PFC from anterior to posterior (A to P) sections in excitatory (left) and inhibitory (right) neurons. **c**, Spatial organization of L4/5 IT 1 and L5 ET 1 as examples from A to P sections. **d**,**e**, Violin plots showing the cortical depth distributions of excitatory neuron subtypes (**d**) and inhibitory neuron subtypes (**e**) in PFC (cell number = 121,617). The maximum cortical depth is normalized to 1. **f**, Spatial location of five representative neuron subtypes (excitatory neuron subtypes: L2/3 IT 2, L5 ET 1, L5/6 NP; and inhibitory neuron subtypes: Lamp5 3 and Pvalb 3) in PFC on a coronal slice. Red dots mark the indicated cell types and gray dots mark the other cells. Source data

Mapping the MERFISH clusters onto the sequential anterior–posterior (A–P) sections revealed the order of cellular organization in 3D throughout the sections (Fig. 2a). Heterogeneous distribution of several neuron subtypes along the A–P and dorsal–ventral (D–V) axes was visually evident. Analysis along the A–P axis revealed that L2/3 IT and L4/5 IT neuron subtypes are enriched in the anterior-most part, where all types of L5 and L6 neurons are generally low (Fig. 2b). This density gradient follows a reverse order in the posterior direction where deep layer neurons like L5 ET 1 or L6 CT 1–3 are gradually enriched (Fig. 2b). Detailed mapping of various neuron subtypes on the serial brain sections clearly revealed the uneven distribution along the A–P axis (Fig. 2c and Extended Data Fig. 6b,c). In contrast, some subtypes such as L5/6 NP are modularly distributed and few others (for example, L5 IT 2 or L6 IT 1) are sparse, but uniform throughout the A–P axis (Fig. 2b).

There is also strong distribution heterogeneity among the inhibitory neurons, but it follows a pattern of regional enrichment instead of gradual transitions along the A–P axis (Fig. 2b). For some subtypes, such as Lamp5 3, Pvalb 4 and Vip 2, the fluctuation in density along the A–P axis is very prominent (Fig. 2b). Neighborhoods with high density of distinct interneuron subtypes may indicate regulatory hotspots or focal points for specific subcortical projections circuits.

**Extended Data Fig. 7:**
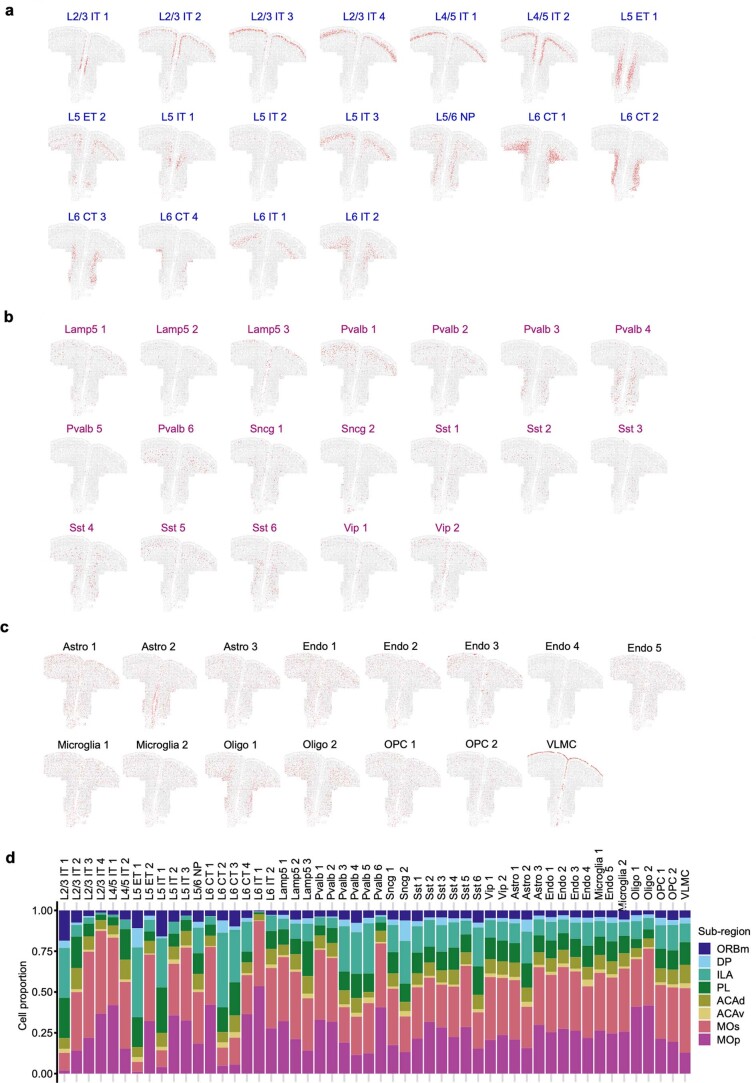
Spatial location of all molecularly defined PFC cell types and subtypes. **a**, Excitatory neuron subtypes; **b**, inhibitory neuron subtypes; **c**, non-neuron cell types and subtypes. Red dots represent the indicated cell types and subtypes. **d**, The proportion of cell numbers from different PFC subregions and adjacent cortical regions of all neuron and non-neuron subtypes. Source data

Another readily recognizable feature from the coronal slices is the laminar organization of various excitatory neurons along the D–V axis, within each representative section (Fig. 2a). Computation of physical depth inward from the cortical surface revealed that IT neurons located more superficially within each layer. The L2/3 IT (and L4/5 IT) subtypes are most superficial and closer to the surface of the brain (Fig. 2d). Similarly, in L5, most IT neurons (L5 IT 1, L5 IT 3) are superficial to the other populations of the layer (L5 ET 1, L5 ET 2) (Fig. 2d). Within layer 6, although L6 IT 1 is superficial, L6 IT 2 mingles with the deepest CT subtypes (Fig. 2d). Plotting each population individually onto a representative coronal section clearly resolved a highly specific spatial localization of each neuron subtype in layers inwards from the cortical surface (Fig. 2f and Extended Data Fig. 7a).

The D–V organization of GABAergic interneurons was even more interesting. Although inhibitory neurons, unlike the excitatory, are not organized in layers, most subtypes appear to be enriched within specific excitatory layers or subregions (Fig. 2e). Broadly, the Lamp5 (Lamp5 1 to 3) and Vip (Vip 1 and 2) neurons along with Sncg 1 are more enriched in superficial layers. Lamp5 3, for example, is restricted only to the superficial layer (Fig. 2e,f). However, Sncg 2 is broadly distributed along the entire depth (Fig. 2e and Extended Data Fig. 7b). This appears to be different from the neighboring motor cortex, as per recent reports^27^, where all subtypes of Sncg neurons are present only in superficial layers. Additionally, the motor cortex also has some subtypes of Vip neurons in deeper layers, which was not detected in PFC. However, the most interesting observation is that specific molecular subtypes of Pvalb and Sst neurons are differentially enriched in various layers along the cortical depth (Fig. 2e). For example, while Pvalb 5 and Pvalb 2 have higher density toward the superficial layers, Pvalb 3 and Pvalb 6 are enriched in the very deep layers, and Pvalb 1 and Pvalb 4 are maximally enriched in the intermediate region (Fig. 2e,f and Extended Data Fig. 7b). Likewise, Sst 1 and Sst 5 are more superficially enriched, and the remaining are distributed in the intermediate to deep layers (Fig. 2e and Extended Data Fig. 7b).

Most non-neuronal subtypes displayed a more broad and dispersed distribution (Extended Data Fig. 7c), with few exceptions. The VLMC, for example, line the outermost surface along the cortex. Oligo 1 and Oligo 2 are enriched near the regions of origin of the white matter tracts (Extended Data Fig. 7c). The Astro 2 had a significant presence in L1 and somewhat greater enrichment in the medial prefrontal region (Extended Data Fig. 7c).

### Distinct neuron subtypes are selectively enriched in mouse PFC

**Fig. 3:**
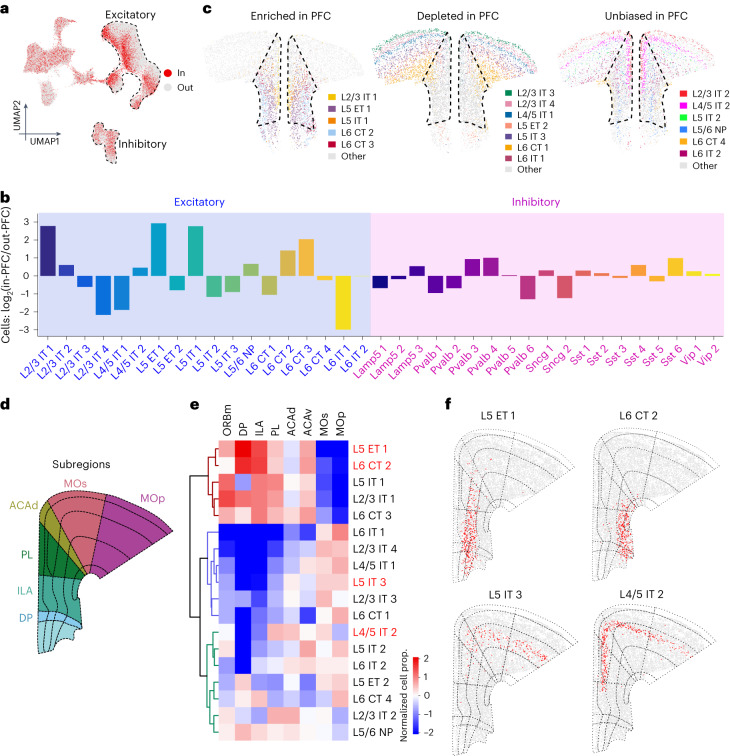
Distinct neuron subtypes are selectively enriched or depleted in PFC relative to the adjacent cortical regions. **a**, UMAP of all MERFISH cells colored by their spatial location whether in or out of PFC (in = 121,617 cells; out = 285,445 cells). **b**, Barplot showing the log_2_ of the abundance ratio of subtype neurons in or out of PFC. **c**, Spatial location of excitatory neuron subtypes enriched (left), depleted (middle) and unbiased (right) distribution in PFC compared with adjacent regions. The dotted line marks PFC in the slice. **d**, Diagram of anatomical subregions of PFC and adjacent cortical regions. **e**, The normalized neuron proportion of excitatory subtypes in different anatomical subregions. **f**, Spatial location of four representative excitatory neuron subtypes on a coronal slice. Red dots represent the indicated subtypes. The dotted lines indicate the anatomical subregions from Allen Brain Atlas CCF v3. Source data

PFC is very distinct in function and connectivity compared to the adjacent cortices. We asked whether this functional and connectivity distinction is associated with its specialized cell composition. To this end, we identified the adult mouse PFC boundary in each section by aligning with CCF v3 (Extended Data Fig. 6a). By projecting the cells identified from the alignment as ‘in’-PFC onto the combined UMAP of the frontal cortex (Fig. 3a), we found that some subtypes of excitatory neurons are selectively biased ‘in’, and some others ‘out’ of the defined PFC region (‘out’ being mainly primary and secondary motor cortices; Fig. 3a), indicating different cellular composition in PFC and the adjacent areas. Calculation showed that L2/3 IT 1, L5 ET 1 and L5 IT 1 are about eightfolds enriched within the PFC, whereas L6 CT 2 and L6 CT 3 are more than twofolds (Fig. 3b). In contrast, L2/3 IT 4, L4/5 IT 1 or L6 IT 1 are markedly depleted (fourfolds to eightfolds) in the PFC (Fig. 3b). When mapped onto the representative coronal section, the enriched, depleted and unbiased populations were clearly visible with respect to the boundaries of the PFC (Fig. 3c). Inhibitory neurons, although less abundant, exhibit clear subtype selectivity across all the major types in PFC (Fig. 3b). Switching of Pvalb subtypes (~2-fold enriched in Pvalb 3 and 4, and depleted of Pvalb 1, 2 and 6), depletion of Sncg 2 and enrichment of Sst 4 and Sst 6 are the most prominent features (Fig. 3b and Extended Data Fig. 7b). Notably, Lamp5 3, the most superficially located interneuron (L1) is the only enriched Lamp5 neuron in PFC (Fig. 3b). The relative proportions of specific IT, ET and CT subtypes are intimately tied to the projections of a cortical area (inside and outside the telencephalon). The selection of specific interneurons determines the precise excitatory–inhibitory balance in the input/output circuits of the projections. In combination, these circuit motifs likely serve as a blueprint for the specialized functions of a specific cortical area, and PFC is clearly organized into a highly selective assembly in this regard.

The PFC has distinct functional subregions from its dorsal to ventral end, viz. dorsal anterior cingulate cortex (dACC or ACAd), prelimbic cortex (PL), infralimbic cortex (ILA), dorso-peduncular cortex (DPP; Fig. 3d) and also part of the medial orbitofrontal cortex (ORBm), present more anteriorly. We asked whether these subregions have distinct cellular composition. Indeed, clustering with the normalized cell proportions across all subregions revealed the most enriched excitatory neurons in each subregion (Fig. 3e, Extended Data Fig. 7d). For example, L5 ET 1 is enriched in PL and ILA (but depleted in ACAd), while L6 CT 2 is mainly in ILA and L5 IT 3 is mainly in ACAd (Fig. 3e,f, enriched cells in panel e are labeled by red fonts). In ORBm, L2/3 IT 1 is enriched, but L2/3 IT 4 of the same layer is strongly depleted (Fig. 3e). The differential neuron subtype distribution in the different PFC subregions can help explain PFC’s subregion-specific functions (how some subregions regulate specific behaviors) and their implications in physiological and pathological complexity of neuropsychiatric processes.

### Distinct transcriptional signatures emerge in mouse PFC

**Fig. 4:**
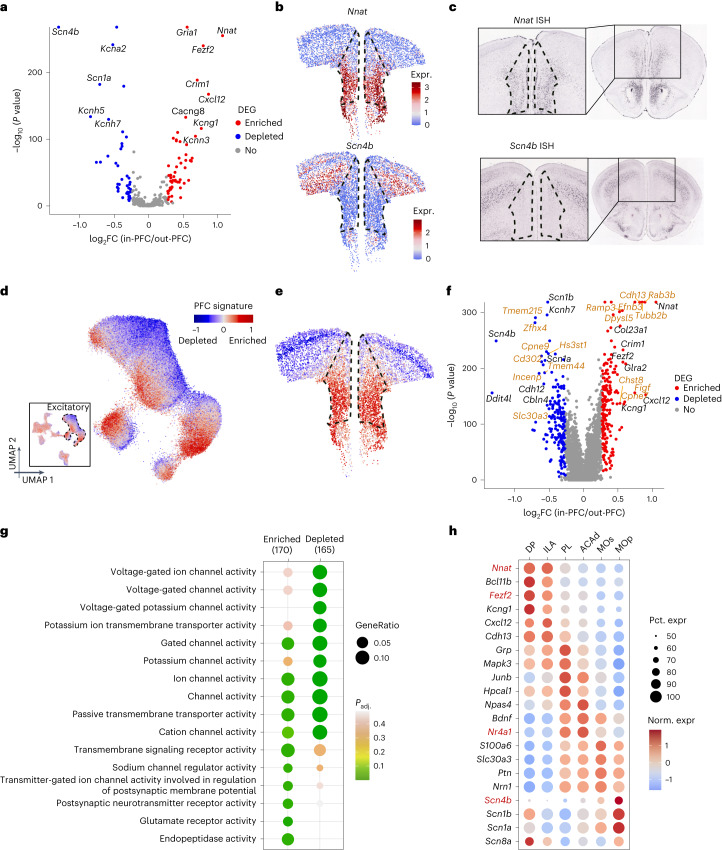
Genes with expression enriched or depleted in PFC. **a**, Volcano plot showing the DEGs that are enriched or depleted in PFC neurons relative to the neurons out of PFC (in = 121,617 cells; out = 285,445 cells). Expression of genes enriched, depleted in PFC, are colored in red and blue dots, respectively (two-sided Wilcoxon test, Bonferroni corrections for multiple comparison; genes with adjusted *P* < 0.01 and fold change > 1.2 defined significant). **b**, Spatial gene expression of *Nnat* (top) and *Scn4b* (bottom) in all excitatory neurons. The dotted line marks PFC region. **c**, ISH data from Allen Brain Atlas showing the spatial expression of *Nnat* and *Scn4b* in a coronal slice (right) with zoom-in (left). **d**, UMAP of all MERFISH cells (bottom-left) and excitatory neurons colored by the PFC signature, which is defined as the average expression of top ten enriched genes minus the average expression of top ten depleted genes. **e**, Aligning the PFC signature onto a representative slice to show the spatial distribution of PFC signature. **f**, Volcano plot showing the expressions of genes enriched or depleted in PFC after imputing by iSpatial. A total of 20,733 genes are analyzed. Genes analyzed by MERFISH are colored in black, and genes inferred by iSpatial are colored in yellow (two-sided Wilcoxon test, Bonferroni corrections for multiple comparison; genes with adjusted *P* < 0.01 and fold change > 1.2 defined significant) **g**, The gene ontology enrichment analysis of genes that are enriched or depleted in PFC (one-sided Fisher’s exact test, Benjamini–Hochberg method for multiple comparison). **h**, Gene expression enrichment analysis of genes enriched in the different anatomical subregions of PFC and the adjacent cortical regions. Source data

Functional differences across brain regions often underlie molecular adaptations^21^. The cortex is believed to be no exception. Thus, we asked whether the distinctive functions and cellular organization of the PFC are associated with specialized molecular features by comparing the transcriptome of PFC with that of the adjacent cortical regions. Indeed, a large number of genes interrogated in the MERFISH library are differentially expressed between the PFC and the neighboring cortices (Fig. 4a). Among the 416 genes analyzed, 54 were substantially enriched and 40 depleted in PFC (adjusted *P* < 0.01, expression fold change > 20%; Supplementary Table 2). Mapping expression of substantially enriched (*Nnat*) or depleted (*Scn4b*) genes onto the coronal section showed clear enrichment or depletion in the PFC region (Fig. 4b), which is consistent with the ISH data from the Allen Brain Institute (Fig. 4c), validating our MERFISH results.

**Extended Data Fig. 8:**
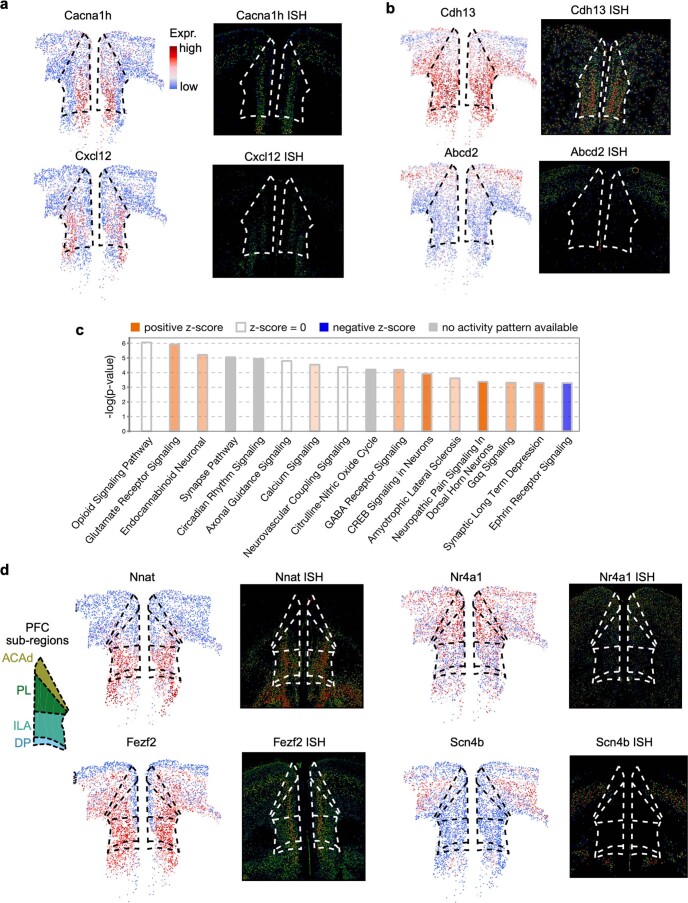
Specific gene expression signatures of PFC and PFC subregions. **a**,**b**, Spatial expression of representative genes enriched (Cacna1h, Cxcl12, Cdh13) or depleted (Abcd2) in PFC relative to adjacent cortical regions. Inferred expression by iSpatial is shown in b. Only excitatory neurons are shown. Corresponding ISH data from Allen Brain Atlas are shown on the right. Dotted line marks PFC region. **c**, Ingenuity pathway analysis (IPA) of the genes, identified after imputation, showing enriched or depleted in PFC. The red/blue bars indicate the pathway more active in/out PFC, respectively (p-values were calculated by One-sided Fisher′s Exact Test without adjustments for multiple comparison). **d**, The inferred spatial gene expression by iSpatial of four representative genes enriched in PFC subregions. A diagram of anatomical subregions in PFC and adjacent regions is shown on the left. Only the excitatory neurons are shown. ISH data from Allen Brain Atlas are shown on the right. Dotted line marks PFC subregion. Source data

Next, we asked whether specific types or categories of genes are selectively enriched or depleted in PFC. The DEGs had a strong representation of several ion channels and some key neurotransmitter receptors, which can impart very distinct electrical properties characteristic of the PFC, relative to adjoining cortices^31^. Several potassium channels are enriched or depleted (Supplementary Table 2). The voltage-gated potassium channels subtypes^31–33^, especially delayed rectifiers (*Kcna2, Kcnb2, Kcnc2, Kcnc3, Kcnq3* and *Kcnq5*) and inward (*Kcnh7)* or outward (*Kcnh5*) rectifier are depleted (Fig. 4a and Supplementary Table 2). BK channel like *Kcnmb4*, reciprocally enriched modifiers/silencers like *Kcng1* or *Kcnf1* and posthyperpolarization regulator like *Kcnn3* are enriched (Fig. 4a and Supplementary Table 2). Mutations of these genes are often implicated in psychiatric disorders (like *Kcnn3* in schizophrenia and bipolar disorder^34^). Apart from potassium, some prominent calcium channels (*Cacna1e* and *Cacna1h*; Extended Data Fig. 8a) and sodium (*Scn3b*) channels, which are implicated in autism and epilepsy^35–38^, are also enriched.

Besides gated ion channels, another striking observation in PFC is the selective enrichment of *Gria1* (Fig. 4a), a principal ionotropic AMPA glutamate receptor subunit (forms dimer–dimer pair with GluA2), which is implicated in several neuropsychiatric disorders (such as schizophrenia, epilepsy and depression), chronic pain (increase), Alzheimer’s (decrease) and drug addiction (decrease)^39,40^. Interestingly, *Cacng8*, a transmembrane AMPA receptor-regulating auxiliary subunit (regulates AMPA trafficking and gating), implicated in psychiatric disorders, is also enriched within PFC (Fig. 4a)^41^. Finally, chemokines like *Cxcl12*, which shape inhibitory neuron synapses to neuro-immune interactions, are also upregulated in PFC (Fig. 4a and Extended Data Fig. 8a)^42–44^.

To globally represent the remarkable transcriptional features of PFC neurons, we calculated the ‘PFC signature’, the average expression of the top ten enriched genes minus the top ten depleted genes. When values for this index were projected (as red color) onto cells in the original UMAP (Uniform Manifold Approximation and Projection, for dimension reduction), the PFC-enriched excitatory neurons clearly clustered and emerged (Fig. 4d). When the PFC signature was mapped onto a representative coronal section, it localized precisely within the anatomical limits of the PFC (Fig. 4e), indicating a distinct molecular composition of the PFC relative to the adjacent cortices.

### iSpatial: transcriptome-wide PFC-enriched genes and pathways in mouse

To expand our spatial mapping of gene expression to the transcriptome scale (including genes beyond the MERFISH library), we integrated our prior PFC scRNA-seq data^17^ and current MERFISH data to predict the expression pattern of all genes using iSpatial^45^, a bioinformatic tool that we developed. The analysis revealed 190 PFC-enriched and 182 PFC-depleted genes (Fig. 4f and Supplementary Table 2). Mapping enriched and depleted candidate genes predicted by iSpatial, *Cdh13* and *Abcd2*, respectively, onto a coronal section revealed consistent localization with respect to the PFC boundaries (Extended Data Fig. 8b), which is similar to Allen Brain ISH results (Extended Data Fig. 8b).

Gene ontology enrichment analysis of the 372 spatially DEGs revealed biological function categories highly enriched in transporters, channels and receptor activity, which are known to modulate membrane potential (Fig. 4g). Depletion of voltage-gated potassium channels or transmembrane potassium transporter concur with a poised state of activity that PFC neurons must maintain for working memory function, a feature not essential for adjacent motor or sensory cortices^33,46^. Greater enrichment of ‘postsynaptic neurotransmitter activity’ or ‘glutamate receptor activity’ (Fig. 4g) relative to adjacent cortices reaffirms that PFC retains substantial plasticity compared to these regions, even in adults. Curiously, some functions such as ‘gated channel’ or ‘cation channel activity’ show enrichment as well as depletion (Fig. 4g). This indicates that PFC likely uses a different subset of receptors (class switching) for the same functions compared to adjacent cortices to adapt to its distinct electrophysiological needs.

A signaling pathways enrichment analysis of these 372 genes revealed opioid signaling, endocannabinoid pathway and glutamate receptor signaling as the top three pathways (Extended Data Fig. 8c). While glutamate signaling is widespread in the cortex, opioid and cannabinoid signaling are more characteristic of the PFC (in mood, memory, feeding, etc.)^47–49^. This indicates that the distinct molecular composition of PFC is indeed tied to its specialized functions.

Decoding the transcriptome-wide, spatially enriched, gene expression patterns also allowed us to investigate whether there is expression bias between subregions of the PFC. Indeed, we detected several genes (for example, *Nnat*, *Fezf2*, *Nr4a1*, *Scn4b*, etc.) that are preferentially expressed in certain subregions of the PFC (Fig. 4h and Extended Data Fig. 8d), which are also validated in Allen Brain ISH data (Extended Data Fig. 8d). Thus, subregion-specific functions of PFC are potentially enabled by discrete molecular compositions imparting specific electrical and signaling properties.

### Organization predicts subtype-specific interactomes in mouse PFC

**Fig. 5:**
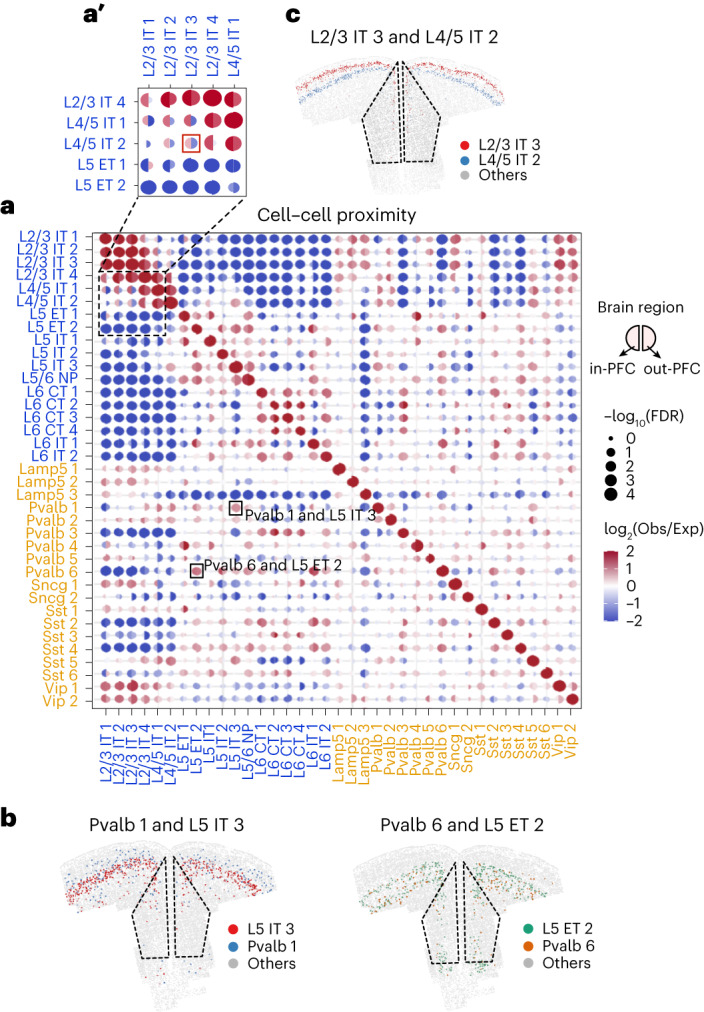
Cell–cell proximity across all neuronal cell types. **a**, Enrichment of cell–cell proximity of different subtypes shown in dot plot (total number of neurons = 32,811). The left half of each dot indicates the cell–cell proximity in PFC and the right half dot indicates that outside the PFC. The color represents log_2_-transformed observation to expectation of colocalized frequency of the two clusters. The size of dots indicates the significance of the colocalization (**a′** shows an enlarged inset highlighting examples of proximities that are different ‘in’ and ‘outside’ of PFC). **b**, A representative slice showing the cell locations of Pvalb 1 and L5 IT 3 neurons (left), and Pvalb 6 and L5 ET 2 neurons (right). **c**, A representative slice showing the cell locations of L2/3 IT 3 and L4/5 IT 2 neurons. The dotted lines mark the PFC region. Source data

PFC integrates multilevel (thalamic, cortical and subcortical) inputs within local circuits for efficient cognitive processing^50–52^. We asked whether potential cell–cell interactions between neuron subtypes can be predicted by the organizational map revealed by MERFISH. We queried the cell subtype composition of the neighborhood of each cell and calculated the enrichments of paired subtype–subtype colocalizations. We also compared the interactions between the in-PFC and out-of-PFC regions, and presented them as two halves of a circle for each interaction (Fig. 5a). We found that enrichment of proximity was notable among many groups of cells (Fig. 5a). For example, first considering the inside PFC alone, the IT subtypes of L2/3 are closely apposed in the superficial layers and potentially engage in cortico-cortical interactions with sensory and association cortices (Fig. 5a). Interestingly, most of these subtypes have interactions with L4/5 IT subtypes (Fig. 5a) that receive exclusive inputs from thalamus or lower order cortex (because PFC has no clear L4), which are known to relay processed information to L2/3 (ref. ^52^). Interestingly, our analysis also revealed specific interactions in the deeper layers that may not be apparent from the histological organization. For example, L6 IT neurons (like L6 IT 1) share proximity with specific ET neurons (L5 ET 2), revealing subtype selectivity (and, in turn, circuit selectivity) within L5-L6 communication (Fig. 5a). Subtype selectivity is perhaps most important in excitatory–inhibitory coupling. Preferential pairing of many excitatory subtypes with one or few (but not all) specific Pvalb subtypes was detected (Fig. 5a). For example, L5 IT 3 scored the highest proximity with Pvalb 1, while L5 ET 2 (located within the same layer) has greater interaction probability with Pvalb 6 (Fig. 5a, highlighted boxes). Mapping cells onto a representative coronal section revealed the relative proximities of each of these two excitatory–inhibitory pairs, and also a different spatial enrichment of the Pvalb 1 and Pvalb 6 subtypes (Fig. 5b). While some proximities like Pvalb 1/L5 IT 3 and Pvalb 6/L5 ET 2 appeared similar both ‘in PFC’ and ‘out of PFC’ (Fig. 5a,b), many other subtypes show either weaker or altogether different cell–cell proximity features inside versus outside of PFC. For example, the L2/3 IT 3 neurons are close to L4/5 IT 2 neurons in the PFC region, but they are located far away outside the PFC region (Fig. 5a,a′,c). Thus, MERFISH allows the prediction of subtype-specific interactions based on spatial organization, which can be systematically studied in the future (by histology and physiology) to identify distinct circuits engaged by specific behaviors.

### Spatial and molecular organization of projections of mouse PFC

It is well known that PFC’s excitatory pyramidal neurons project to different subcortical targets including the striatum, NAc, thalamus, hypothalamus, amygdala, periaqueductal gray (PAG) or ventral tegmental area^16,53^. However, the spatial organization of projection neurons, and whether different neuron subtypes project to different targets, is not well characterized.

**Fig. 6:**
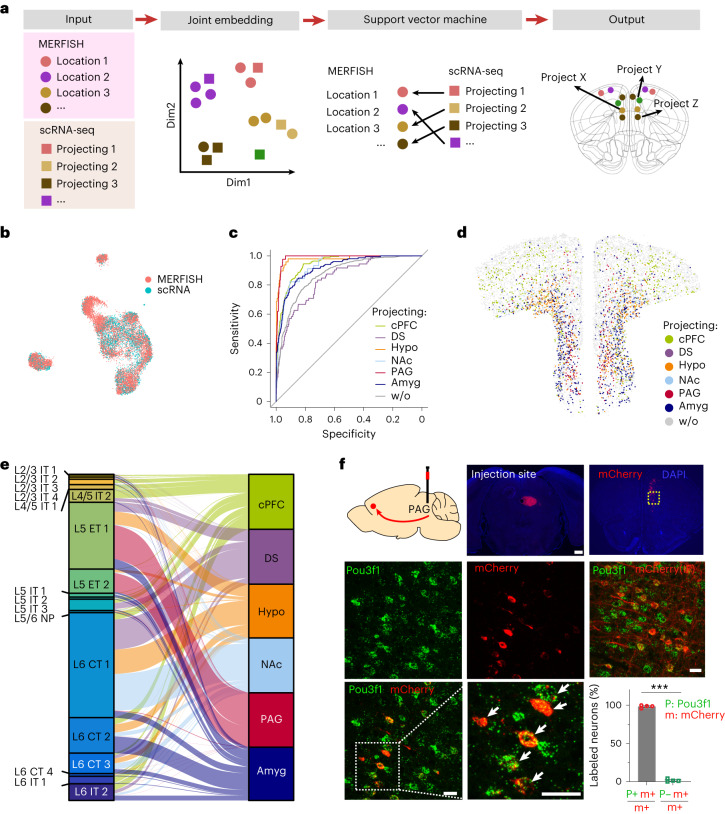
Spatial and molecular organization of PFC projection to the major subcortical targets. **a**, Schematics of the strategy for inferring neuronal projection of MERFISH clusters. The MERFISH and scRNA-seq data are integrated into a reduced dimensional space. An SVM is used to predict neuronal projection of the MERFISH neuron subtypes (Methods). **b**, UMAP visualization of cells derived from MERFISH (9,544 cells) and scRNA-seq (4,294 cells) data after integration. **c**, The ROC curves show the prediction powers of six projection targets; w/o represents the cells without projection information. **d**, A coronal slice showing in silico retrograde tracing from six injection sites, labeled by different colors as indicated. **e**, The inferred projection targets of molecularly defined excitatory neuron subtypes, represented by an alluvial diagram. **f**, PFC to PAG projection validation. Retrograde mCherry-expressing AAV was injected in PAG—injection scheme cartoon and injection site in PAG are shown (scale bar = 0.5 mm); brain slice of PFC was used for smFISH. mCherry (red) labeled neurons coexpressing the L5 ET 1 marker Pou3f1 (green), arrows in the enlarged image indicate double-labeled neurons. Co-immunostaining for mCherry protein with Pou3f1 RNA-FISH further confirmed extensive colocalization. (scale bars = 20 µm). Bar graph shows the percentage of mCherry positive cells that also express Pou3f1 (mean ± s.e.m., two-tailed *t* test, *n* = 4 biologically independent adult male mice, *P* < 0.001). Majority of mCherry^+^ neurons are Pou3f1^+^. Hypo, hypothalamus. Source data

**Extended Data Fig. 9:**
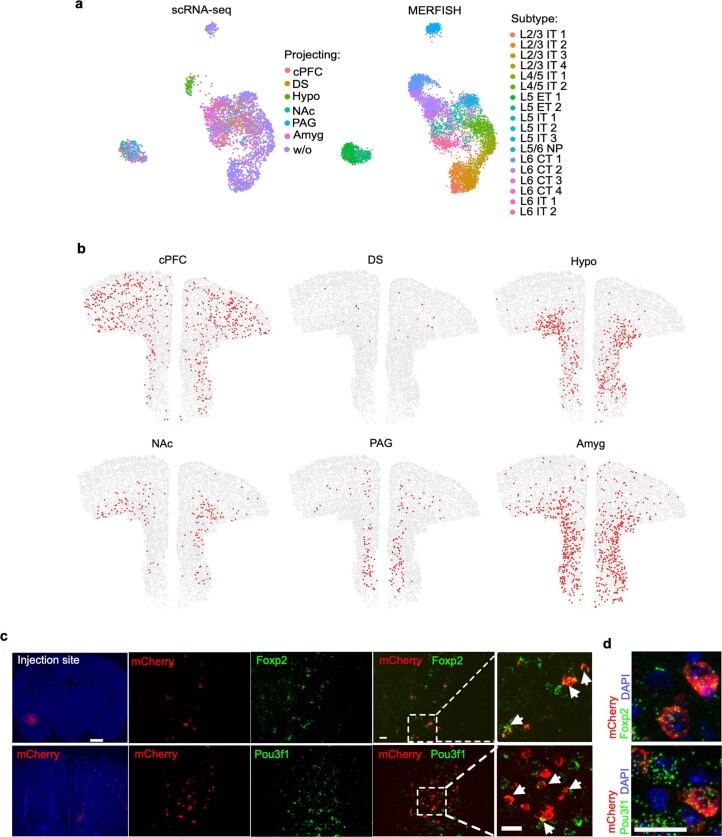
Integrated MERFISH and scRNA-seq data to predict neuronal projections. **a**, UMAP showing integration of cells from scRNA-seq (left) and MERFISH (right). The colors represent the projection sites in scRNA-seq data and the excitatory subtype in MERFISH data, respectively. **b**, Spatial location of neurons projecting to six different brain regions. **c**, Amygdala projection validation: mCherry expressing retrograde AAV was injected in amygdala (injection site shown) (scale = 0.5mm). Brain slice of PFC were stained with DAPI and mCherry to image the labeled neurons. smFISH co-labeling of mCherry with Pou3f1 (L5 ET marker), or Foxp2 (L6 CT marker) reveal partial overlap with both neuron subtypes. (Stains independently repeated 3 times). Arrows in enlarged images indicate dual labeled cells (scale bar = 20µm). **d**, High resolution images showing smFISH co-labeling of mCherry with Foxp2 and Pou3f1 within individual neurons (scale bar = 20µm).

A prior study performed scRNA-seq of PFC neurons retrogradely labeled from some of these major targets^2^. We integrated our PFC MERFISH data with this dataset to predict the PFC neuron subtypes and their spatial/layer location, which project to these different targets. Through joint embedding and supervised machine learning, we could assign respective projection identity to the molecular clusters organized in space within the PFC (Fig. 6a). An overlap of the MERFISH and scRNA-seq clusters through UMAP visualization revealed a strong correspondence (Fig. 6b and Extended Data Fig. 9a). The receiver operating characteristic (ROC) curve for the prediction model independently predicted six different projection targets with high confidence, including contralateral PFC (cPFC), dorsal striatum (DS), hypothalamus, NAc, PAG and amygdala (Fig. 6c). Mapping these projection neurons onto a coronal slice of frontal cortex revealed the identity and spatial organization of neurons that project to each of these six targets within the PFC (Fig. 6d). Distinct spatial localization of each of these six groups of cells can be visualized when mapped individually on the coronal slice (Extended Data Fig. 9b). This analysis allowed us to associate different subsets of each neuronal type that project to different regions with their location within the PFC (Fig. 6e), which reveals that most of the target brain regions receive projection from more than one neuron subtypes. For example, the amygdala receives projections from all four subtypes of L6 CT neurons as well as L5 ET 1 neurons, but the majority comes from L6 CT 2. Likewise, the hypothalamus receives its projections from L5 ET 1 and L6 CT 1; DS from L6 CT 1, 2 and 3; and NAc gets mainly from L6 CT 1, L5 ET 1 and some from L6 CT 2. However, one exception is the PAG, which receives its projections almost exclusively from L5 ET, predominantly from L5 ET 1 (and some from L5 ET 2). Consistent with prior knowledge, superficial layer IT neurons project to the contralateral hemisphere of PFC^54^.

To validate our computational model-based projection prediction, we injected retrograde labeling adeno-associated virus (AAV) (driving mCherry expression) into PAG and amygdala. Four weeks after the injection, single-molecule FISH (smFISH; RNAScope) showed that consistent with the prediction, all mCherry mRNA expressing PFC neurons, retro-traced from PAG, colabeled with *Pou3f1*, a selective marker for L5 ET 1 and L5 ET 2 (Fig. 6f). To further confirm, we also co-immunostained mCherry protein with *Pou3f1* RNA-FISH showing that every mCherry-expressing neuron contained Pou3f1 mRNA (Fig. 6f). In amygdala, colocalization of mCherry was detected for both *Pou3f1* (L5 ET 1) and *Foxp2* (L6 CT) as predicted (Extended Data Fig. 9c). High-resolution confocal images revealed strong mCherry expression in subsets of Foxp2^+^ and Pou3f1^+^ neurons, respectively (Extended Data Fig. 9d). These data support the accuracy of our circuit predications.

### Identifying PFC neuron subtypes involved in chronic pain

The role of PFC in cognition and executive function is most widely studied. However, PFC also has a pivotal role in autonomically modulating pain perception, and aberrations in this process are emerging as a major determinant in pain ‘chronification’^5,12^. While chronic pain is escalating as a leading healthcare challenge^7^, molecular underpinnings of the dysfunction remain unknown. Chronic pain has been strongly associated with transcriptional adaptations across the PFC^5,55^; however, the spatial or cell-type-specific resolution of these changes is less clear. Using MERFISH, we attempted to identify the specific PFC neuron subtypes that are impacted by chronic pain.

**Fig. 7:**
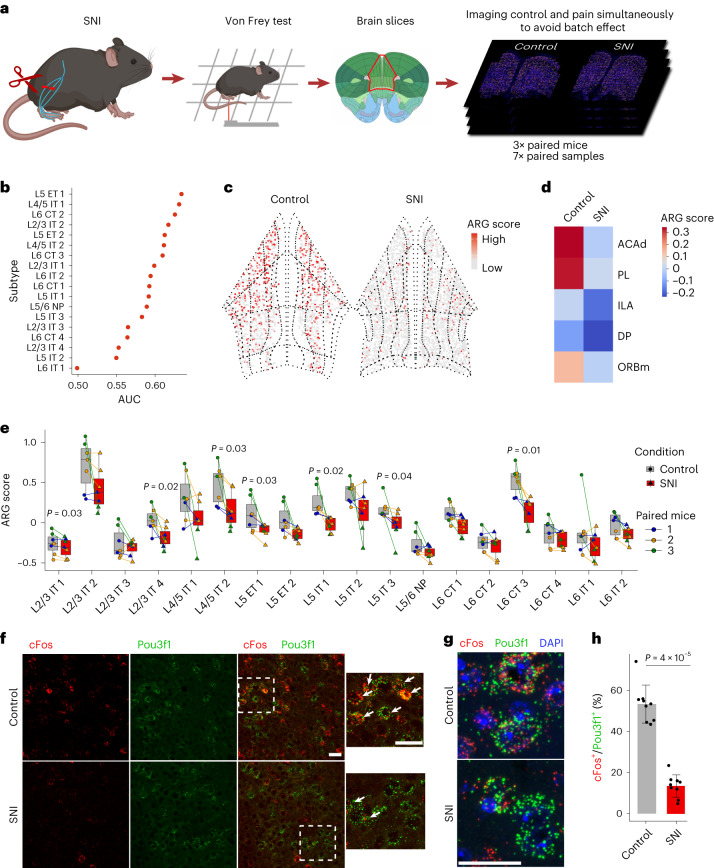
Chronic pain caused cellular and molecular changes in PFC excitatory neurons. **a**, Overview of chronic pain sample preparation. For each MERFISH run, one brain slice from each of the control and pain condition are loaded together to avoid batch effect (*n* = 3 biologically independent adult male mice per group in sham and SNI, sampled over seven sessions of imaging experiments). **b**, Transcriptionally perturbed neurons predicted by Augur for each of the excitatory subtypes. AUC shows the area under the ROC curve of the predictions. **c**, Spatial distribution of cells colored by ARG scores in control and pain conditions. The anatomical subregions of PFC are also shown. **d**, Heatmap showing ARG score in PFC subregions in pain and control samples (number of neurons in **b**–**d**, *n*_sham_ = 17,873, *n*_SNI_ = 19,392). **e**, ARG scores of PFC excitatory subtypes in pain and control samples. Paired dots represent the control–pain paired samples, which were imaged together. Color of the paired dots represents the paired mice ID (two-tailed paired *t* test is used to calculate the *P* values; *n* = 3 independent mice per group; the center is the median value, bounds of box indicate the first and third quantile; the minima are defined as the minimum values and the maxima are defined as maximum values within each group). **f**, smFISH colabeling of cFos and Pou3f1 (L5 ET marker) at high magnification in sham (control) and SNI (chronic pain) conditions. Arrowheads in merged images indicate double positive neurons (scale bars = 20 µm). **g**, High-resolution images showing localization of cFos and Pou3f1 mRNA molecules within individual neurons in sham and chronic pain (scale bar = 20 µm). **h**, Barplot showing the percentage of cFos^+^ cells to Pou3f1^+^ cells (mean ± s.e.m.; two-tailed Mann–Whitney test; *n* = 9 biologically independent adult male mice per group; *P* = 4 × 10^−5^). Source data

**Extended Data Fig. 10:**
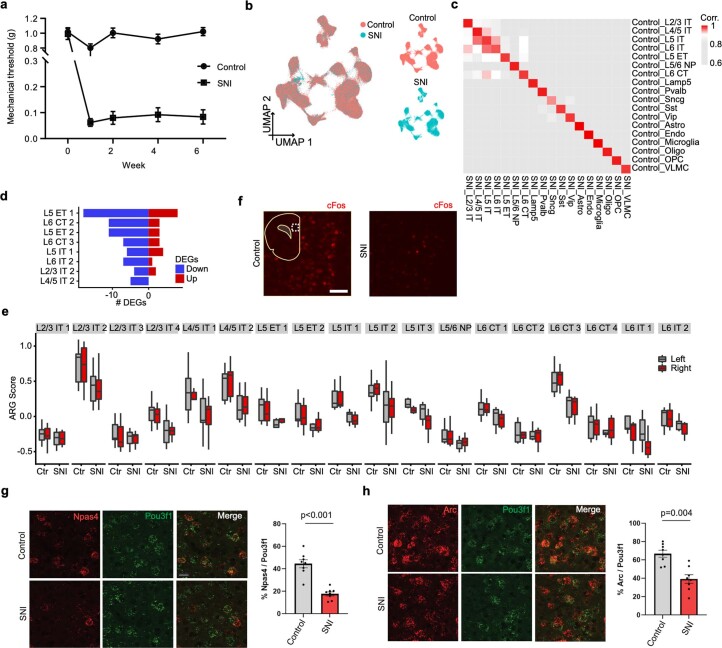
Pain model: Behavior, MERFISH and smFISH measurements. **a**, Von Frey testing reveals chronic mechanical allodynia induced by SNI surgery during the 6 weeks testing period (n = 6 biologically independent adult male mice per group, sham and SNI, measured weekly for 6 weeks). **b**, UMAP visualization of cells from control and pain samples. Left panel: all cells from both groups combined; Right panel: separate view of the control and pain. **c**, Heatmap showing the gene expression correlation of the cell types between control and pain. **d**, The numbers of differentially expressed genes in the indicated neuron subtypes when compared pain and control samples. The numbers of up-regrated and down-regulated genes are colored in red and blue, respectively. **e**, Comparison of ARG scores between the two hemispheres of PFC in each mouse (two-tailed paired t-test is used to calculate the p*-*value; n = 3 biologically independent mice per group; the center is the median value, bounds of box indicate the first and third quantile; the minima are defined as the minimum values and the maxima are defined as maximum values within each group. Cell numbers, sham = 17873, SNI = 19392). **f**, Global overview of PFC in half coronal section with Fos smFISH (red) in Sham (Control) and chronic pain conditions (scale bar = 100µm). **g**, Npas4 and **h**, Arc expression is reduced across PFC in chronic pain relative to control and is significantly lower in Pou3f1+ neurons. (mean ± SEM; Mann-Whitney test; n = 8 biologically independent adult male mice per group of sham and SNI; Npas4, p = 0.0002; Arc, p = 0.004). (scale bar in g = 20µm, for g and h). Source data

To this end, we used the well-established spared nerve injury (SNI) model of chronic neuropathic pain in mice^56^ where two of the three branches of the sciatic nerve are transected (Fig. 7a), which causes a state of chronic neuropathic pain in the hind paw that lasts for months. We performed SNI and sham (control: nerve exposed, but not transected) surgeries in adult male mice and conducted weekly von Frey tests to assess mechanical sensitivity. Strong mechanical allodynia characteristic of neuropathic pain developed in the SNI mice that persisted throughout the 6-week testing period (Extended Data Fig. 10a). Six weeks after surgery, brains from three pairs of sham and SNI mice were collected and characterized with MERFISH (Fig. 7a). UMAP visualization and overlap (Extended Data Fig. 10b), followed by transcriptomic cell-type comparisons (Extended Data Fig. 10c) affirmed high correlation and convergence of sham and SNI datasets ensuring a reliable comparison to reveal the effect of chronic pain.

We employed Augur^57^ to identify the cell type(s) that are transcriptionally most perturbed by chronic pain, which revealed L5 ET 1 as the most affected subtype followed by the L4/5 IT 1, L6 CT 2 and L5 ET 2 (Fig. 7b). The most number of DEGs were also detected in L5 ET 1 followed by L6 CT 2, L5 ET 2 and few others (Extended Data Fig. 10d). No significant changes were detected in the other 30 clusters despite many of the excitatory neuron subtypes being highly abundant in PFC, suggesting that these clusters are minimally affected in chronic pain. Interestingly, the two most impacted clusters, respectively, project to PAG (L5 ET 1) and amygdala (L6 CT 2; Fig. 6e), the two major hotspots known to regulate sensory and affective aspects of pain^5,58^.

Chronic pain is known to inflict strong and sustained hypoactivity across the PFC^9,10,12,59^. We asked whether this can be detected in the baseline expression of neuronal ARGs to identify prominently affected neuron subtypes. We calculated the ARG score using the mean expression of a panel of five ARGs (*Arc*, *Junb*, *Fos*, *Npas4* and *Nr4a1*) and compared between sham and SNI groups (Methods). We observed a strong and widespread reduction of ARG score when it is plotted on representative coronal sections (Fig. 7c). A subregion-specific analysis revealed that the ACAd and PL are the most impacted PFC subregions (Fig. 7d). We next compared the differences of ARG score across the individual excitatory neuron clusters (Fig. 7e), and found that it is downregulated in several clusters, including those exhibiting transcriptional changes (for example, L5 ET 1 and L6 CT 3; Extended Data Fig. 10d). We also examined whether there is a biased decline in ARG score between the two PFC hemispheres of individual mice (Extended Data Fig. 10e). Despite some trends, no significant difference was detected, potentially indicating that prolonged chronic pain states (like 6 weeks here) can trigger more widespread impact in brain. Stronger ipsilateral versus contralateral bias may be evident during the early stages of pain ‘chronification’ and remains an interesting subject of future study.

To validate chronic pain-induced hypoactivity across PFC, we performed smFISH to compare Fos expression between sham and SNI brain sections. Although sham shows a baseline Fos activity in PFC, a general decrease in Fos signal is obvious in the SNI (Extended Data Fig. 10f). Costaining Fos with Pou3f1, a selective marker for L5 ET 1, revealed significant Fos downregulation in this neuron subtype in the SNI brains (Fig. 7f–h). At high resolution, strong differences in RNA counts can be clearly visualized in single neurons (Fig. 7g). While Fos is commonly used, we employed two more ARGs for validation, Npas4 and Arc, both of which are reduced in PFC, particularly in L5 ET 1 neurons labeled by Pou3f1 (Extended Data Fig. 10g,h). Thus, the PAG projecting Pou3f1 neurons underwent one of the strongest reductions of ARG activity score, indicating potential impact on its baseline electrical activity.

Despite the conventional knowledge that a PFC–PAG circuit is involved in the descending modulation of pain^5^, its cell-type identity or changes in chronic pain were unclear. Our findings revealed the molecular identity and spatial organization of this circuit—the L5 ET 1 neurons with PAG projection (Fig. 6e), which underwent strong reduction of ARG activity score in chronic pain (Fig. 7f,g) and also incurred the most transcriptional perturbation (Fig. 7b). Additionally, we also identified at least two CT subtypes in L6 (L6 CT 2 and 3) that project to limbic structures such as amygdala, NAc and hypothalamus (Fig. 6e) that may be involved in the affective response to pain.

## Discussion

In this study, we present an account of how the PFC is distinctly organized at the molecular, cellular and projection levels relative to the adjacent regions within the frontal cortex. We exploit these features to reveal the molecular identity of key neuron subtypes that are engaged in chronic pain, and more broadly, we provide a foundation for systematic mapping of functional ensembles and circuits selectively engaged in various cognitive and executive functions of PFC. Spatial transcriptomics is a rapidly developing field^60^, and similar to recent studies^25,27,61^, MERFISH enabled systematic decoding of PFC’s molecular and cellular organization.

The cellular composition of a cortical area should be dictated by the input and output circuits associated with its function. We observed that a variety of neuronal subtypes are specifically enriched in PFC (Fig. 3a–c). This regional subtype specificity potentially underlies the characteristic properties of the PFC relative to other cortical regions. For example, the PFC is agranular and lacks a typical L4 (associated with thalamic input), it receives long-range inputs across all of its layers and projects to subcortical targets from different layers, and engages in reciprocal circuits with most of these targets^16,62^. The twofold enrichment of the superficial-most IT neurons (L2/3 IT 1) likely facilitates cortico-cortical communications, but the subsequent IT populations (L2/3 IT 4 or L4/5 IT 1) are markedly depleted to likely make room for more L5 IT 1 or L5 ET 1 that engage in long-distance subcortical projections. Enrichment of two CT subtypes (L6 CT 2 and 3) is consistent with the observation that CT neurons of PFC project to several subcortical targets (Fig. 6e), rather than thalamus alone. Notably, two of these enriched neuron subtypes (L5 ET 1 and L6 CT 2) emerge as key players in chronic pain, a function majorly assigned to the PFC within the cerebral cortex (Fig. 7). Inhibitory cell composition also has very substantial implications. For example, depletion of certain subtypes of Pvalb (Pvalb 1, 2 and 6), also resulting in an overall lower count of Pvalb neurons in the PFC (relative to the adjacent regions), suggests that feedforward inhibition (and hence excitatory/inhibitory balance) is differently organized in PFC. This is an important observation because functional imbalance of Pvalb neurons has been implicated in almost every PFC-associated disease, such as schizophrenia^63^, bipolar, depression and chronic pain^64^. Detection of all these regional differences would not be possible without spatial profiling techniques like MERFISH.

Besides cellular composition, we detected strong transcriptional features (especially with ion channels or receptors) specific to the PFC compared to adjacent cortices (Fig. 4). It is generally appreciated that different cortical regions have different baseline electrical properties and qualitatively different activity patterns, which in turn is critical for their specific function^21^. Recording of electrical field potentials across cortical areas provides strong evidence supporting such regionally variable activity patterns^65,66^. However, the biological/molecular bases of such functional differences have been less clear. Our findings revealing preferential expression or repression, or even subtype switch of a wide range of ion channels, and key glutamate receptor subunits in PFC demonstrate potential mechanisms underlying regionally specific electrical properties in the cortex.

We identified the key cell types in PFC that are specifically impacted by chronic pain (Fig. 7). Amidst the rising prevalence of chronic pain and emerging consensus that transition to chronic pain is centrally regulated, there has been little clarity about the cellular and circuit mechanisms underlying the ‘chronification’, which is key to therapeutic targeting. Previous studies have shown that transcranial stimulation of PFC could relieve chronic pain^9,11,13,67^. Such studies, although established a causal connection, provide limited long-term solutions for pain management owing to the potentially deleterious effects of broad nonspecific cortex-wide stimulations. Despite a long-standing general knowledge of putative PFC to PAG projections in descending inhibition of pain^5^, the molecular identity of this circuit was unknown. In this regard, our finding of L5 ET 1 as a major neuron subtype exclusively projecting to the PAG, and undergoing transcriptional changes in chronic pain state, is of particular relevance. While the reduced activity of L5 ET 1 can impair descending inhibition to potentiate physical pain, it remains to be determined whether it also contributes to the affective component of pain. However, L6 CT 2 and L6 CT 3, the other two implicated clusters, project to multiple limbic regions including amygdala, NAc and hypothalamus, and their dysfunctions may elicit strong negative affect characteristic of chronic pain states^58,68^. All these remain valuable prospects for future functional studies through targeted neuronal activity manipulation using genetically engineered animal models.

Leveraging the resolution of MERFISH, this study revealed a wealth of information about the distinct cellular, molecular and circuit organization, as well as functional properties of the mouse PFC. However, some limitations remain, which are as follows: (1) despite better resolution in clustering (than scRNA-seq), characterization of inhibitory neuron function remained limited. While the biological replicates per group (*n* = 3) in MERFISH are sufficient to support the current findings, a larger number of mice or more sensitive techniques in the future may resolve this issue and clarify the role of inhibitory neurons in chronic pain; (2) although MERFISH can achieve very efficient single-cell resolution, it is restricted to a fixed library of preselected genes. Accordingly, studies are limited to hypotheses, and new transcriptional genome-wide changes cannot be discovered for which other lower-resolution spatial techniques can be integrated into future studies and (3) while ARGs provide an effective means to determine the activity history of neurons and help narrow down causal subtypes, physiological slice recordings of these cells (from sham and SNI mice) in future would be necessary to provide the ultimate proof for neuronal activity changes under chronic pain.

## Methods

### Mice and surgery

All experiments were conducted in accordance with the National Institute of Health Guide for Care and Use of Laboratory Animals and approved by the Institutional Animal Care and Use Committee (IACUC) of Boston Children’s Hospital and Harvard Medical School. Wildtype male C57BL6 mice of about 10 weeks old were used for all experiments in the study. Mice were maintained at 12-h light/12-h dark cycles with food and water ad libitum. For the SNI surgery, mice were anesthetized with ketamine. Hair was shaved above the knee on one side (usually left) and the skin was sterilized with iodine and isopropanol. The muscles were separated by blunt dissection to expose all three branches of the sciatic nerve. The tibial and common peroneal branches of the nerve that run parallel were tied tightly with two sutures and a piece between the two ties was transected and removed. Care was taken that the third branch (sural nerve) was untouched during the whole procedure. The retracted muscles were released, and the skin was stitched back. In the sham surgery group, identical steps were followed to expose the nerve, but no transection was performed, and skin was stitched back in position. Six weeks after the surgery, brains were collected to assess the impact of chronic pain. Mice were tested the day before being harvested to confirm ongoing mechanical allodynia. On the day of harvest, any acute handling was avoided, and SNI/sham mice were taken directly from their home cage to euthanasia and brains were immediately harvested and frozen (within 5–7 min). These brains were eventually sectioned at 14-μm thickness to collect samples along the A–P axis and subjected to MERFISH.

### MERFISH library design and encoding probes

The MERFISH library of 416 genes belongs to the following three categories: (1) cell-type markers; (2) neuronal function regulatory genes and (3) neuronal ARGs. Cell-type markers were determined based on our previous bulk and single-cell sequencing of the PFC that were used to distinguish different cell subtypes and subtypes, with priority for neurons (major cell-type markers, subtype markers and cortical layer markers). The functional genes are comprised of genes regulating neural activity and function such as ion channels, receptors and neuropeptides. We started with a comprehensive list of all ion channels, receptors and peptides reported in cortex^21^ and then refined to only those expressed in PFC^17^. Finally, based on previous studies^25^, we selected a panel of neuronal ARGs whose expression can report the activity history of neurons.

A library of MERFISH encoding probes for all target genes was generated as described previously^25^. Briefly, a unique binary barcode was assigned to each gene based on an encoding scheme with 24 bits, a minimum Hamming distance of 4 between all barcodes, and a constant Hamming weight of 4. This barcoding scheme left 60 ‘blank’ barcodes unused to serve as a measure of false-positive rates. For each gene, 50 to 70 30-nt-long targeting regions with limited homology to other genes and narrow melting temperature and GC ranges were selected, and individual encoding probes to that gene were created by concatenating two 20-nt-long readout sequences to each of these target regions. Each of the 24 bits was associated with a unique readout sequence, and encoding probes for a given gene contained only readout sequences for which the associated bit in the barcode assigned to that gene contained a ‘1’. Template molecules to allow the production of these encoding probes were designed by adding flanking PCR primers, with one primer representing the T7 promoter. This template oligopool was synthesized by Twist Biosciences and enzymatically amplified to produce encoding probes using published protocols^25^.

### MERFISH tissue processing and imaging

Tissue was prepared for MERFISH as described previously^25^. Briefly, mice were killed under CO_2_ and brains were quickly harvested and rinsed with ice-cold calcium and magnesium-free PBS. The brains were frozen on dry ice and stored at −80 °C till sectioning. The frozen brains were embedded in OCT on a mixture of ethanol and dry ice. Serial 14-μm-thick frontal cortex sections spaced about 150 μm apart were collected and placed on poly-d-lysine coated, silanized coverslips containing orange fiducial beads prepared as described previously^25^. The sections were allowed to briefly air dry and immediately fixed with 4% PFA for 10 min. Sections were washed in PBS and stored in 70% ethanol for at least 12 h to permeabilize. The sections were washed in hybridization buffer (2× SSC + 30% formamide) and then drained and inverted over parafilm in petri dish onto a 50 μl droplet of mixture containing encoding probes and a poly(A) anchor probe^25^ in hybridization buffer (2× SSC, 30% formamide, 0.1% yeast tRNA and 10% dextran sulfate) and hybridized in a covered humid incubator at 37 °C for 2 d. Coverslips were then washed in hybridization buffer and the sections were embedded into a thin film of poly-acrylamide gel, as described previously. The embedded sections were then digested for 2 d in a 2× SSC buffer containing 2% SDS, 0.5% Triton X-100 and 1:100 proteinase K. The coverslips were washed and stored in 2× SSC at 4 °C until imaging. MERFISH imaging was performed on a custom microscope and flow system, as described previously^25^. In each imaging round, the volume of each slice was imaged by collecting a *z* stack at each field-of-view containing 10 images each spaced by 1 μ. Twelve imaging rounds using two readout probes per imaging round were used to read out the 24-bit barcodes. Readout probes were synthesized by Biosynthesis and contained either a Cy5 or Alexa750 conjugated to the oligonucleotide probe via a disulfide bond, which allowed reductive cleavage to remove fluorophores after imaging, as described previously. A readout conjugated to Alexa488 and complementary to a readout sequence contained on the polyA anchor probe was hybridized with readouts associated with the first two bits in the first round of imaging.

### Image processing, decoding and cell segmentation

MERFISH data were decoded as previously described^25^. Briefly, images of fiducial beads collected for each field-of-view in each imaging round were used to align images across imaging rounds. RNAs were detected using a pixel-based approach, in which images were first high-pass filtered, deconvolved and low-pass filtered. Differences in the brightness of different imaging rounds were corrected by an optimized set of scaling values, determined from an iterative process of decoding performed on a randomly selected subset of fields-of-view, and the intensity trace for individual pixels across all imaging rounds was matched to the barcode with the closest predicted trace as judged via a Euclidean metric and subject to a minimum distance. Adjacent pixels matched to the same barcode were aggregated to form putative RNAs. RNA molecules were then filtered based on the number of pixels associated with each molecule (greater than 1) and their brightness to remove the background.

As described previously^25^, the identification of cell boundaries within each field of view (FOV) was performed by a seeded watershed approaching using DAPI images as the seeds, and the poly(A) signals to identify segmentation boundaries. Following segmentation, individual RNA molecules were assigned to specific cells based on localization within the segmented boundaries.

### Preprocessing of MERFISH data

The decoded data were preprocessed by the following steps: (1) segmented ‘cells’ with a cell body volume less than 100 µm^3^ or larger than 4,000 were removed; (2) cells with total RNA counts of less than 10 or higher than 98% quantile, and cells with total RNA features less than 10, were removed; (3) to correct for the minor batch fluctuations in different MERFISH experiments, we normalized the total RNA counts per cell to a same value (500 in this case); (4) doublets were removed by Scrublet^69^ and (5) the processed cell-by-gene matrix was transferred to gene-by-cell matrix and then loaded into Seurat V4 (ref. ^70^) for downstream analysis. The matrix was log-transformed by the Seurat standard pipeline.

### Cell clustering

Two rounds of cell clustering were used to identify cell types and subtypes. In the first round, we identified the following three major cell types: excitatory neurons, inhibitory neurons and non-neuronal cells. In the second round, each major cell type was further clustered. Excitatory neuron was further clustered into 18 subtypes, inhibitory neurons was further clustered into 19 subtypes and non-neuronal cell was further clustered into 15 subtypes. Then, we separated the excitatory subtypes into the following seven groups according to the neuronal projection: L2/3 IT, L4/5 IT, L5 IT, L6 IT, L5 ET, L5/6 NP and L6 CT. The inhibitory neuron was cataloged into the following five groups based on the main markers: Lamp5, Pvalb, Sncg, Sst and Vip. Non-neuronal cells were cataloged into the following six groups: endothelial cells, microglia, oligodendrocytes, OPC, astrocytes and VLMC. Each round of clustering follows the same workflow as described previously. First, all gene expression was centered and scaled via a *z* score, and PCA was applied to the scaled matrix. To determine the number of principal components (PCs) to keep, we used the same method described before^25,61^. Briefly, the scaled matrix was randomly shuffled and PCA was performed based on the shuffled matrix. This shuffling step was repeated 10 times, and the mean eigenvalue of the first principal component crossing the 10 iterations was calculated. Only the PCs derived from the original matrix that had an eigenvalue greater than the mean eigenvalue were kept. Harmony^71^ was then used to remove the apparent batch effect among different MERFISH samples. The corrected PCs were used for cell clustering. The nearest neighbors for each cell were then computed by a K-nearest neighbor (KNN) graph in corrected PC space. Bootstrapping was used for determining the optimal *k* value for KNN as previously described^25,61^ (*k* = 10 in the first round clustering. *k* = 50, 20 and 15 for excitatory neurons, inhibitory neurons and non-neuronal cells, respectively, in the second round). Leiden method was used for detecting clusters^72^. The resolution was set to 0.3 in the first round of clustering and was set to 2 for the second round. Finally, we manually removed the clusters representing doublets, which express high levels of the established markers of multiple cell types. Clusters located outside of the cortex were also removed.

### Correspondence between scRNA-seq and MERFISH clusters

To compare the cell clusters identified by scRNA-seq and MERFISH, we first co-embedded the two datasets in a corrected PCA space using Harmony as described above. Then, all the cells from both scRNA-seq and MERFISH were used to build the KNN graph. The first 30 corrected PCs were inputted into Seurat::FindNeighbors to compute the KNN. For each cell cluster in MERFISH, we obtained the cell cluster’s nearest 30 neighbor cells’ information. Then, we calculated the percentages of the cell clusters derived from scRNA-seq that were near this MERFISH cluster, from which we obtained a correspondence matrix, where each row is a cluster from scRNA-seq, each column is a cluster from MERFISH and the element in the matrix indicates the similarity between the two clusters. Similarly, for each cell cluster in scRNA-seq, we inquired about the nearest clusters derived from MERFISH data to generate another correspondent matrix. The average of the two correspondent matrices was used to indicate the similarities between the cell clusters defined by scRNA-seq and MERFISH.

### Cortical depth measurement

After MERFISH cell segmentation, the cells’ spatial location was determined by their centroid coordinates. For each cell in PFC region, the cortical depth was measured as the shortest spatial distance between the cell location and the cortical surface line. VLMC cells are a monolayer located on the cortical surface and used to label the cortical surface in each MERFISH slice.

### Imputing transcriptome-wide expression by iSpatial

MERFISH reveals gene expression and location at single-cell resolution but only targets 416 predefined genes. We used iSpatial (version 1.0.0) to infer the transcriptome-wide spatial expression. In short, iSpatial co-embed MERFISH and single-cell RNA-seq datasets by two sequential rounds of integration. The scRNA-seq data are from our prior PFC scRNA-seq data^17^. For each cell of the MERFISH dataset, iSpatial searches for the nearest neighbors from scRNA-seq data by a weighted *k*-nearest neighbors model. Then, the expression values of the neighbors are assigned to the MERFISH data, resulting in a transcriptome-wide spatial expression profile.

### Detecting neuronal subtype or gene expression enriched/depleted in the PFC

For each brain slice, we first normalized the total number of cells detected for each neuronal subtype, as the selected PFC adjacent regions for MERFISH imaging showed little difference in different slices. The ratio of the normalized cell number in the PFC to that outside the PFC was calculated in each neuronal subtype. Then, the ratio was log_2_ transformed and used to indicate the subtype enrichment/depletion in the PFC.

To detect the differently expressed genes between the cells in the PFC and adjacent regions, the Wilcoxon rank test was applied to each gene. Then Bonferroni correction was used to adjust the *P* value for multiple comparisons. Seurat FindAllMarkers was used for this analysis.

### Gene ontology enrichment analysis and ingenuity pathway analysis (IPA)

Based on transcriptome-wide spatial expression that was inferred by iSpatial^45^, the DEGs between the PFC and adjacent regions were calculated and then used for gene ontology enrichment analysis and IPA. The compareCluster function from clusterProfiler (version 4.0.2)^73^ was used for gene ontology enrichment analysis. All the inferred genes were chosen as the background list. IPA software (version Spring Release, April 2022)^74^ was used for IPA. IPA not only identifies the most significant pathways but also the pathways predicted to be activated or inhibited based on the input gene list. IPA calculates the following two different statistics: (1) for the *P* value, IPA uses a Fisher’s exact test to calculate the likelihood of the overlap between the input genes and the known pathways. The significance indicates the probability of association of input genes with the pathway by random chance alone; (2) for the *z* score, IPA considers the directional effect (activation or inhibition) of the genes’ expression changes on a pathway.

### ARG score calculation

The ARG score is calculated based on the expression of the following five neuronal activity-related genes^75^: *Arc, Junb, Fosb, Npas4* and *Nr4a1*. Because the baseline expression of these genes is different, we first *z* scored the expression by genes to standardize the expression. The *z* score is calculated by its expression in each cell subtracting the mean expression level and then dividing by the standard deviation of that gene across all cells. For each cell, we calculated the ARG score by averaging the *z* scores of the five neuronal activity-related genes.

### Cell–cell proximity

For each cell, we first identified the nearest 30 neighbors based on spatial distance. Next, we derived the cell subtypes of these neighboring cells and obtained the cell subtypes composition of these cells near the inquired cell. After iteration of all cells in all subtypes, we could calculate the number of occurrences of paired cell–cell and obtain the cell–cell proximity matrix (observed matrix). Because of the cell number differences for each subtype, we normalized cell–cell proximity matrix by a random shuffled matrix (expected matrix). To derive the shuffled matrix, we first shuffled the cell identities by randomly assigning a subtype for each cell. Then, the random cell–cell proximity matrix was calculated by the same method before. Finally, the normalized cell–cell proximity matrix was calculated by log_2_(observed matrix/expected matrix). In addition, the *P* values were calculated by Wilcoxon rank tests (using wilcoxon.test in R) and then adjusted by Benjamini–Hochberg method (using p.adjust in R, method = ‘BH’).

### Excitatory neuron projection prediction

The scRNA-seq data (GEO: GSE161936)^2^ were first preprocessed by standard Seurat pipeline. Only the cells from dorsomedial (dmPFC) and ventromedial (vmPFC) regions were used. We integrated the MERFISH and scRNA-seq data using Harmony, and all the cells derived from MERFISH/scRNA-seq were co-embedded on a corrected PCA space. The first corrected 30 PCs were selected as features to train a multiclass support vector machine (SVM) for predicting the neuronal projection. The cells from scRNA-seq were separated into training and test groups. Then, the SVM was trained on training data and validated on test data by using the radial basis function kernel. Gamma was set to 0.01, and cost was set to 10. The ROC curve was plotted to evaluate the performance using pROC package^76^ in R. Finally, the model was applied to MERFISH cells to predict their projections, and the area under the curve (AUC) was equal to 0.913.

### Register MERFISH slice to Allen Brain Atlas

To align MERFISH slices to the Allen Brain Atlas CCF v3, we leveraged the spatial distribution of cells identified by MERFISH in each slice as well as DAPI images of that slice. First, each brain slice was paired to the closest matching coronal section in CCF v3 with the help of DAPI image and spatial location of the cell types. Then, we modified the WholeBrain package^77^ to align the MERFISH slice to the corresponding matching CCF coronal section. To ensure accurate alignment, we leveraged the MERFISH cell typing result at single-cell resolution and used certain cell types as anchors to help locate the anatomic features. VLMC cells are used for marking the surface of brain slice as follows: inhibitory neuron subtype, Lamp5 3, for locating layer 1, L2/3 IT neurons for locating layer 2, L6 CT neurons for locating layer 6 and oligodendrocytes for locating corpus callosum. Because some small slices do not have sufficient features to align, 45 of 60 slices are successfully registered to CCF v3, which allowed us to define the anatomic PFC and PFC subregions.

### Testing mechanical allodynia in SNI mice

Mechanical allodynia due to SNI neuropathy was tested in sham and SNI mice using von Frey monofilaments. Animals were placed in testing room to habituate for 30 min. After 30 min, mice were placed in enclosures (Ugo Basile, 37000-006) on a perforated metal platform (Ugo Basile, 37450-005) with opaque walls separating mice for an additional 30 min to habituate. We employed the widely used up-down method for testing, as originally described in ref. ^78^. Starting with the base filament, higher or lower filaments were consecutively applied till a response was documented—followed by the series of four filaments to document response patterns, based on which the paw withdrawal threshold was calculated^78^.

Mice were tested the day before surgery to ensure a uniform baseline sensitivity across cohorts. SNI surgery was performed the following day. After 7 d, the mechanical sensitivity was similarly tested every seventh day up to sixth week. On the day after the final test, mice were killed and brains were collected for MERFISH.

### DEGs between pain and control conditions

To detect DEGs and correct the batch effects, we used a logistic regression framework. For each gene, we constructed a logistic regression model to predict the sample conditions *C* by considering the batch information S, *C* ~ *E* + S, and compared it with a null model, *C* ~ 1 + S, with a likelihood ratio test. Then, the Bonferroni correction method was applied to adjust for multiple comparisons. Here ‘LR’ method in Seurat FindAllMarkers was used for conducting this analysis.

Sample sizes and statistical tests for all experiments in this manuscript were determined based on established literature in the field from us and others who have reported single-cell transcriptomics, viral tracing and pain assays. Moreover, samples were randomized, and data collection was blinded during experiments wherever appropriate to avoid any skewing or bias in data collection. No data points were excluded.

### Reporting summary

Further information on research design is available in the Nature Portfolio Reporting Summary linked to this article.

## Online content

Any methods, additional references, Nature Portfolio reporting summaries, source data, extended data, supplementary information, acknowledgments, peer review information, details of author contributions and competing interests and statements of data and code availability are available at 10.1038/s41593-023-01455-9.

## Supplementary information

Reporting Summary

Supplementary Table 1 List of MERFISH probes.

Supplementary Table 2 List of enriched and depleted genes in PFC compared to the adjacent cortical regions.

Supplementary Table 3 List of genes whose expression is affected by chronic pain.

## Source data

Source Data Fig. 1 Statistical source data.

Source Data Fig. 2 Statistical source data.

Source Data Fig. 3 Statistical source data.

Source Data Fig. 4 Statistical source data.

Source Data Fig. 5 Statistical source data.

Source Data Fig. 6 Statistical source data.

Source Data Fig. 7 Statistical source data.

Source Data Extended Data Fig. 1 Statistical source data.

Source Data Extended Data Fig. 2 Statistical source data.

Source Data Extended Data Fig. 3 Statistical source data.

Source Data Extended Data Fig. 5 Statistical source data.

Source Data Extended Data Fig. 7 Statistical source data.

Source Data Extended Data Fig. 8 Statistical source data.

Source Data Extended Data Fig. 10 Statistical source data.

## Data Availability

The MERFISH data generated in this study has been deposited to Zenodo: 10.5281/zenodo.8247792. Interactive visualization of MERFISH data can be accessed at https://yizhang-lab.github.io/PFC. Public datasets are used in this study. Single cell RNA-seq of PFC is available at https://www.ncbi.nlm.nih.gov/geo/query/acc.cgi?acc=GSE124952 and https://portal.brain-map.org/atlases-and-data/rnaseq/mouse-whole-cortex-and-hippocampus-smart-seq. MERFISH of mouse motor cortex is available at 10.35077/g.21. MERFISH of mouse frontal brain is available at https://cellxgene.cziscience.com/collections/31937775-0602-4e52-a799-b6acdd2bac2e. MERFISH of human STG and MTG is available at 10.5061/dryad.x3ffbg7mw. Source data are provided with this paper.

## Code availability

Code for analyzing and generating figures is available at GitHub: https://github.com/YiZhang-lab/PFC-MERFISH and Zenodo: 10.5281/zenodo.8247763.
