# Author Correction: Spatial transcriptomics reveals the distinct organization of mouse prefrontal cortex and neuronal subtypes regulating chronic pain

**DOI:** 10.1038/s41593-025-02077-z

**Published:** 2025-09-16

**Authors:** Aritra Bhattacherjee, Chao Zhang, Brianna R. Watson, Mohamed Nadhir Djekidel, Jeffrey R. Moffitt, Yi Zhang

**Affiliations:** 1 Howard Hughes Medical Institute, Boston Children’s Hospital, Boston, MA USA; 2 Program in Cellular and Molecular Medicine, Boston Children’s Hospital, Boston, MA USA; 3 Division of Hematology/Oncology, Department of Pediatrics, Boston Children’s Hospital, Boston, MA USA; 4 Department of Microbiology, Harvard Medical School, Boston, MA USA; 5 Center for Applied Bioinformatics, St. Jude Children’s Research Hospital, Memphis, TN USA; 6 Department of Genetics, Harvard Medical School, Boston, MA USA; 7 Harvard Stem Cell Institute, Boston, MA USA

**Keywords:** Molecular neuroscience, Cellular neuroscience

Correction to: *Nature Neuroscience* https://www.nature.com/articles/s41593-023-01455-9, published 16 October 2023.

In the version of the article originally published, in Extended Data Fig. 10g, the Pou3f1 Control and SNI images were in reverse order. The figure has been amended in the HTML and PDF versions of the article.

