# Supplementary material for: Spatial transcriptomics reveals the distinct organization of mouse prefrontal cortex and neuronal subtypes regulating chronic pain
Source: Nat Neurosci. 2023 Oct 16;26(11):1880–93. doi: 10.1038/s41593-023-01455-9 (PMC10620082; doi:10.1038/s41593-023-01455-9)
Supplement: Supplementary file 1 — Reporting Summary [file 41593_2023_1455_MOESM1_ESM.pdf]

nature portfolio

Corresponding author(s): Yi Zhang

Last updated by author(s): Aug 26, 2023

Reporting Summary

Nature Portfolio wishes to improve the reproducibility of the work that we publish. This form provides structure for consistency and transparency in reporting. For further information on Nature Portfolio policies, see our Editorial Policies and the Editorial Policy Checklist.

Statistics

For all statistical analyses, confirm that the following items are present in the figure legend, table legend, main text, or Methods section.

n/a	Confirmed	
☐	☒	The exact sample size (*n*) for each experimental group/condition, given as a discrete number and unit of measurement
☐	☒	A statement on whether measurements were taken from distinct samples or whether the same sample was measured repeatedly
☐	☒	The statistical test(s) used AND whether they are one- or two-sided<br>*Only common tests should be described solely by name; describe more complex techniques in the Methods section.*
☒	☐	A description of all covariates tested
☒	☐	A description of any assumptions or corrections, such as tests of normality and adjustment for multiple comparisons
☐	☒	A full description of the statistical parameters including central tendency (e.g. means) or other basic estimates (e.g. regression coefficient) AND variation (e.g. standard deviation) or associated estimates of uncertainty (e.g. confidence intervals)
☐	☒	For null hypothesis testing, the test statistic (e.g. *F*, *t*, *r*) with confidence intervals, effect sizes, degrees of freedom and *P* value noted<br>*Give P values as exact values whenever suitable.*
☒	☐	For Bayesian analysis, information on the choice of priors and Markov chain Monte Carlo settings
☒	☐	For hierarchical and complex designs, identification of the appropriate level for tests and full reporting of outcomes
☐	☒	Estimates of effect sizes (e.g. Cohen's *d*, Pearson's *r*), indicating how they were calculated

Our web collection on statistics for biologists contains articles on many of the points above.

Software and code

Policy information about availability of computer code

Data collection	The mouse frontal cortex was collected and imaged by MERFISH to get single-cell level, spatial transcriptome of the cells in the cortex.
Data analysis	The MERFISH data were analyzed by Seurat (v 4.0.5). Harmony (1.0) was used for batch correction. iSpatial (v 1.0.0) was used for data imputation.<br>Code for MERFISH analysis is available at Github: https://github.com/YiZhang-lab/PFC-MERFISH and Zenodo: https://doi.org/10.5281/zenodo.8247763.

For manuscripts utilizing custom algorithms or software that are central to the research but not yet described in published literature, software must be made available to editors and reviewers. We strongly encourage code deposition in a community repository (e.g. GitHub). See the Nature Portfolio guidelines for submitting code & software for further information.

Data

Policy information about availability of data

All manuscripts must include a data availability statement. This statement should provide the following information, where applicable:

- Accession codes, unique identifiers, or web links for publicly available datasets
- A description of any restrictions on data availability
- For clinical datasets or third party data, please ensure that the statement adheres to our policy

The MERFISH data generated in this study has been deposited to Zenodo: https://doi.org/10.5281/zenodo.8247792. Interactive visualization of MERFISH data can be accessed at: https://yizhang-lab.github.io/PFC.

Research involving human participants, their data, or biological material

Policy information about studies with human participants or human data. See also policy information about sex, gender (identity/presentation), and sexual orientation and race, ethnicity and racism.

Reporting on sex and gender	N/A
Reporting on race, ethnicity, or other socially relevant groupings	N/A
Population characteristics	N/A
Recruitment	N/A
Ethics oversight	N/A

Note that full information on the approval of the study protocol must also be provided in the manuscript.

Field-specific reporting

Please select the one below that is the best fit for your research. If you are not sure, read the appropriate sections before making your selection.

☒ Life sciences ☐ Behavioural & social sciences ☐ Ecological, evolutionary & environmental sciences

For a reference copy of the document with all sections, see nature.com/documents/nr-reporting-summary-flat.pdf

Life sciences study design

All studies must disclose on these points even when the disclosure is negative.

Sample size	Sample sizes were determined based on standards reported in established literature in the field. Always at least 3 (for sequencing) and above (for all other experiments).
Data exclusions	None
Replication	All experiments were at least duplicated, mostly triplicated and ensured for consistency of results.
Randomization	Randomization was applied wherever appropriate in imaging, injection and behavior. For example, serial sections were randomly imaged on different days
Blinding	MERFISH is all automated, so blinding does not apply. But blinding was implemented in smFISH, image counting and behavior acquisition.

Reporting for specific materials, systems and methods

We require information from authors about some types of materials, experimental systems and methods used in many studies. Here, indicate whether each material, system or method listed is relevant to your study. If you are not sure if a list item applies to your research, read the appropriate section before selecting a response.

Materials & experimental systems

n/a	Involved in the study
☐	☒ Antibodies
☒	☐ Eukaryotic cell lines
☒	☐ Palaeontology and archaeology
☐	☒ Animals and other organisms
☒	☐ Clinical data
☒	☐ Dual use research of concern
☒	☐ Plants

Methods

n/a	Involved in the study
☒	☐ ChIP-seq
☒	☐ Flow cytometry
☒	☐ MRI-based neuroimaging

Antibodies

Antibodies used	pab rabbit-anti mCherry (Abcam-ab167453), immunostaining: dilution 1/500<br>donkey anti-rabbit Alexa 555 Fluor conjugated secondary antibody: dilution 1/1500
Validation	Has been widely used and reported

Animals and other research organisms

Policy information about studies involving animals; ARRIVE guidelines recommended for reporting animal research, and Sex and Gender in Research

Laboratory animals	Male C57BL6 mice were used in this study
Wild animals	No wild animals were used
Reporting on sex	Male mice used- No evidence or indication for sex to be a significant variable between similar groups
Field-collected samples	No field samples were collected
Ethics oversight	All experiments were conducted in accordance with the National Institute of Health Guide for Care and Use of Laboratory Animals and approved by the Institutional Animal Care and Use Committee (IACUC) of Boston Children's Hospital and Harvard Medical School under the HCCM (Harvard Center for Comparative Medicine)

Note that full information on the approval of the study protocol must also be provided in the manuscript.
